# Tuning the Selectivity of the Hydrogenation/Hydrogenolysis of 5‐Hydroxymethylfurfural under Batch Multiphase and Continuous‐Flow Conditions

**DOI:** 10.1002/cssc.202200503

**Published:** 2022-06-28

**Authors:** Daily Rodríguez‐Padrón, Alvise Perosa, Lilia Longo, Rafael Luque, Maurizio Selva

**Affiliations:** ^1^ Dipartimento di Scienze Molecolari e Nanosistemi UniversitàCa' Foscari di Venezia 30123 Venezia Italy; ^2^ Grupo FQM-383 Departamento de Química Orgánica Universidad de Cordoba, Edificio Marie Curie (C-3), Ctra Nnal IV-A, Km 396 14001 Cordoba Spain; ^3^ Scientific Center for Molecular Design and Synthesi of Innovative Compounds for the Medical Industry People's Friendship University of Russia (RUDN University), 6 Miklukho Maklaya st. 117198 Moscow Russia

**Keywords:** 5-hydroxymethylfurfural, biomass valorization, continuous flow, multiphase systems, sustainability

## Abstract

The hydrogenation/hydrogenolysis of 5‐hydroxymethylfurfural (HMF) has been carried out either under single (aqueous) phase or batch multiphase (MP) conditions using mutually immiscible aqueous/hydrocarbon phases, 5 % Ru/C as a catalyst, and both with and without the use of trioctylmethyl phosphonium bis‐(trifluoro methane) sulfonimide ([P_8881_][NTf_2_]) as an ionic liquid (IL). Alternatively, the hydrogenation of HMF was explored in the continuous‐flow (CF) mode with the same catalyst. By changing reaction parameters, experiments were optimized towards the formation of three products: 2,5‐bis(hydroxy methyl)furan (BHMF), 2,5‐bis(hydroxymethyl)tetrahydrofuran (BHMTHF), and 1‐hydroxyhexane‐2,5‐dione (HHD), which were obtained in up to 92, 90, and 99 % selectivity, respectively, at quantitative conversion. In particular, the single (aqueous) phase reaction of HMF (0.2 m) carried out for 18 h at 60 °C under 30 bar of H_2_, allowed the exclusive synthesis of BHMF from the partial (carbonyl) hydrogenation of HMF, while the MP reaction run at a higher *T* and *p* (100 °C and 50 bar) proved excellent to achieve only HHD derived from a sequence of hydrogenation/hydrogenolysis. It is worth noting that under MP conditions, the catalyst was perfectly segregated in the IL, where it could be recycled without any leaching in the aqueous/hydrocarbon phases. Finally, the hydrogenation of HMF was explored in a H‐Cube® flow reactor in the presence of different solvents, such as ethyl acetate, tetrahydrofuran, and ethanol. At 100 °C, 50 bar H_2_, and a flow rate of 0.1 mL min^−1^, the process was optimized towards the formation of the full hydrogenation product BHMTHF. Ethyl acetate proved the best solvent.

## Graphical abstract

**Choose your path**: Two strategies are explored for the upgrading of 5‐hydroxymethylfurfural (HMF) by hydrogenation/hydrogenolysis in batch multiphase and continuous‐flow systems. To the best of our knowledge, the present work constitutes the first example on the synthesis of 1‐hydroxyhexane‐2,5‐dione employing an ionic liquid‐assisted multiphase system, not only reaching high conversion and selectivity but also allowing catalyst/product separation.

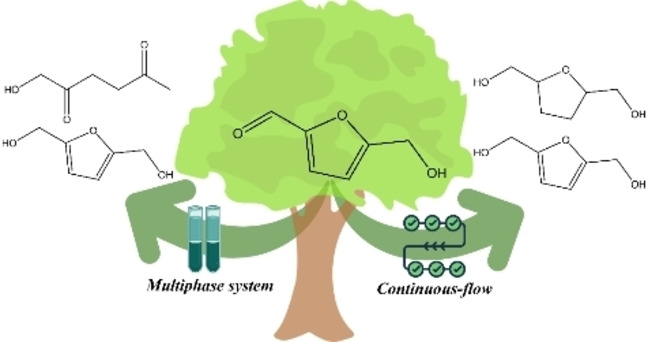

## Introduction

In the past two decades, a massive research effort has been focused on the design of sustainable protocols and technologies for the conversion of biomass into fuels, energy, and chemicals as an alternative to conventional derivatives of fossil origin. Among renewable resources including hydroelectric, solar, geothermal, and wind, biomass is not only the most abundant one but is able to provide both energy and molecules as building blocks for a variety of applications such as plastics, fibers, solvents, fine chemicals, pharmaceuticals, and so on. This potential, however, is still highly underutilized considering that natural photosynthesis allows the growth of 170 billion metric tons of biomass per year, mostly comprised of carbohydrates (75 %), of which only 3–4 % is used by humans.[^1^, ^2^]

**Scheme 1:**
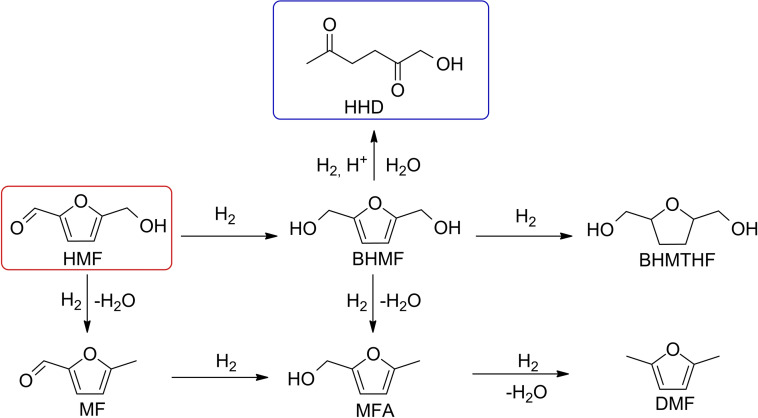
Structure of major hydrogenation and hydrogenolysis products from HMF.

Techno‐economic assessments have demonstrated that the complexity of biomass makes the technological progress of the sector very challenging. The competitiveness of any strategy must therefore ground on multiple‐output biorefining units where the biofuel production is integrated with the synthesis of “high‐value low‐volume” products. In general terms, this implies the conversion of biomass into a range of derivatives, from bulk compounds (e. g., for bioenergy uses) up to specialty chemicals.[^3^, ^4^, ^5^, ^6^] Fundamental in this scenario is the role of the starting feedstocks and the need of robust criteria for their choice. In this paper, according to the Top 10 list of renewable building blocks defined by the Bozell‐Petersen analysis, the focus has been placed on bio‐based furanics, more specifically on the most representative member 5‐hydroxymethylfurfural (HMF). The compresence of multiple sites/functionalities such as the hydroxy group, the aldehyde group, and the aromatic furan ring, makes HMF a versatile substrate with a synthetic and market potential in the field of biofuels, fuel additives, pharmaceuticals, polymers, resins, and solvents.[^7^, ^8^] Such interest has been confirmed by more than 10000 scientific articles and patents on reactivity and applications of this molecule, with approximately 1000 papers published annually in the past decade.^[9]^ In this context, a prominent place for the chemical upgrading of HMF is held by the reactions of hydrogenation and hydrogenolysis. These processes may lead to several products, such as 2,5‐dimethylfuran (DMF), methylfurfural (MF), 2,5‐bis(hydroxymethyl)furan (BHMF), 2,5‐bis(hydroxymethyl)tetrahydrofuran (BHMTHF), and 1‐hydroxyhexane‐2,5‐dione (HHD) (Scheme 1),[^10^, ^11^, ^12^, ^13^, ^14^, ^15^] whose distribution is primarily steered by the nature of catalysts, the temperature, and the pressure.

Among homogeneous catalysts, the most‐used ones include Ru, Ir, or Rh complexes with bidentate N‐heterocyclic carbenes (NHCs), primary amine moieties, and gold sub‐nanoclusters, while heterogeneous systems are often based on supported metals, often in nanoparticle shape, as Pd, Pt, Ru, Ni on C, Pd on SiO_2_, Al_2_O_3_, or TiO_2_. For such systems, typical operating temperature and pressure for the selective conversion of HMF to BHMF are in the range of 50–80 °C and 10–50 bar, respectively,[^15^, ^16^, ^17^, ^18^, ^19^] whereas the ring hydrogenation of BHMF to obtain BHMTHF requires harsher conditions with an increase of either the temperature up 100 °C or the pressure to 80 bar.[^20^, ^21^]

On the other hand, further catalyst design is necessary to obtain linear diols and triols from the hydrogenolysis of HMF. In this sense, several examples have been described in the literature; for instance, the formation of (i) 1,2,6‐hexanetriol (64.5 % yield) was reported over a Ni−Co‐Al mixed oxide catalyst at 120 °C and 40 bar of H_2_,^[22]^ and (ii) 1,6‐hexanediol (43 % yield) was obtained using Pd/zirconium phosphate (ZrP) as a catalyst with formic acid as a hydrogen source and (140 °C, 1 atm).^[23]^

Hydrogenolysis processes may take place competitively to hydrogenation reactions of HMF, if an acidic environment is provided. DMF was obtained in 85–100 % yields using either 5 wt % Pd/C under a combined atmosphere of CO_2_ (100 bar) and H_2_ (10 bar) at 80 °C,^[24]^ or a bimetallic catalyst comprised of a Lewis‐acidic Zn^II^ and Pd/C at 150 °C and 22 bar of H_2_.^[25]^ MF was, instead, prepared in a 80 % yield from HMF using carbon‐supported Pd nanoparticles and formic acid as a hydrogen donor at 200 °C under 5 bar of N_2_.^[26]^

Additionally, an emerging product from the hydrogenolysis pathways of HMF is HHD. Results and conditions of some representative protocols for the transformation of HMF to HHD are summarized in Table 1.

**Table 1:** Conversion of HMF to HHD.

Entry	Catalyst	*T* [ °C]	*p* [bar]	HHD^[a]^ [%]		Ref.
				**Yield**	**Sel**.	
1	Pd/Nb_2_O_5_	140	40	68	73	[27]
2	Ir‐complex^[b]^	120	5	84	84	[28]
3	Pd/C	120	30^[c]^	77	77	[29]
4	Rh−Re/SiO_2_	120	80	81	81	[30]
5	Pd/MIL‐101(Cr)	140	40	82	83	[31]
6	Ir‐complex^[b]^	120	10	76 (69)^[d]^	76	[32]

[a] Yield and selectivity of HHD determined by GC analyses. [b] Bipyridine coordinated Cp‐Ir^III^ half‐sandwich complex (Cp=1,2,3,4,5‐pentamethylcyclopenta‐1,3‐diene) was the catalyst. [c] An additional pressure of CO_2_ (10 bar) was used. [d] Isolated yield.

In spite of the remarkable yields and selectivity observed by gas chromatography (GC), the purification of the product was tricky. The best result so far was very recently claimed by de Vries and co‐workers, who designed a procedure for the isolation HHD in a 69 % (isolated) yield after the reaction of aqueous HMF catalyzed by the half‐sandwich [Cp*Ir(dpa)Cl]Cl (dpa=dipyridylamine) complex, at 120 °C and 10 bar of H_2_.^[32]^ Results led to conclude that the conversion of HMF to HHD still represents a largely unexplored area, and effective synthetic methods remain a major challenge.

Among catalysts for the hydrogenation/hydrogenolysis of HMF, ruthenium‐based systems, more often heterogenous ones, have received special attention. In a seminal work comparing the performance of Ru, Pd, and Pt supported on a variety of materials such as ceria, magnesia‐zirconia, γ‐alumina, carbon, and silica, Ru/CeO_*x*_ and Ru/Mg−Zr emerged as the best systems: at 130 °C and 30 bar of H_2_, in a 2 : 1 biphasic 1‐butanol/water batch reactor, HMF was quantitatively converted, yielding either its partially hydrogenated derivative (BHMF) or its fully hydrogenated derivative (BHMTHF) with 94 and 91 % selectivity, respectively.^[33]^

Even more interesting are carbon‐supported metal catalysts, which, thanks to their low cost, high surface area, chemical inertness, and thermal stability in non‐oxidizing atmospheres, are often the best choice for hydrogenation/hydrogenolysis reactions.^[34]^ A screening investigation of a series of such (commercial C‐supported) catalysts including 5 wt % Pd/C, Pt/C, Rh/C, Ru/C, and Raney‐Ni proved that the Ru was by far the most effective metal for the hydrogenolysis of HMF: at 200 °C and 20 bar of H_2_, in a THF solution, complete conversion was achieved with 95 % selectivity towards the DMF product.^[35]^ By contrast, the amount of DMF ranged from 9 to 16 % using other catalysts. Another recent work compared the performance of Ru/C, Pd/C, and Pt/C with low metal loading (1 wt %) in the reaction of aqueous solutions of HMF (2–3 wt %) under H_2_ pressure.^[36]^ Notably, authors were able not only to tune the conditions towards the selective hydrogenation (with no side‐hydrogenolysis) of HMF, but to demonstrate that Ru/C was the most effective catalyst to this purpose. Optimized yields of 93 % for BHMF and 95 % for BHMTHF were obtained at 50 °C and 30 bar H_2_ and at 100 °C and 50 bar H_2_, respectively.

In light of this analysis, as a part of our interest in both multiphase (MP) and continuous‐flow (CF) protocols for the chemical upgrading of bio‐based molecules,[^37^, ^38^] we were prompted to explore the applicability of such techniques in the HMF context. Batch MP systems has been extensively reported for the hydrogenolysis/hydrogenation processes of a variety of renewable feedstocks including bio‐oils, glycerol, sorbitol, xylitol, and levulinic acid, just to name a few;^[39]^ however, such MP conditions have been seldom described for reactions of HMF. To the best of our knowledge, besides the above‐mentioned example involving a 1‐butanol/water mixture,^[33]^ only one other work claimed the use of a water/cyclohexane biphase for synthesis of BHMTHF with a Ru/SiO_2_ catalyst. A tandem sequence took place through the in‐situ conversion of fructose to HMF followed by its subsequent hydrogenation to BHMTHF.^[40]^

On the other hand, although several protocols have been described for the CF hydrogenation of HMF over variety of catalytic systems,[^41^, ^42^, ^43^, ^44^, ^45^, ^46^, ^47^, ^48^] the design of new CF procedures in this area remains a thrilling option to implement process intensification strategies, address the reaction scale‐up, and improve the productivity.

Considering the aforementioned premises, the work report herein is focused on the development of two novel MP and CF methods based on Ru/C as a catalyst, by which not only an excellent control of the products distribution was achieved for both the hydrogenation and the hydrogenolysis of HMF, but also an effective catalyst/products separation was obtained. It is worth to highlight that, to the best of our knowledge, this work constitutes the first example on the synthesis of HHD employing an ionic liquid‐assisted MP system. Moreover, by tuning reaction parameters, MP‐batch experiments both with and without the use of trioctylmethyl phosphonium bis‐(trifluoro methane) sulfonimide as an ionic liquid were optimized towards the formation of BHMF, BHMTHF, and HHD in up to 92, 90, and 99 % selectivity, respectively, at quantitative conversion. Additionally, an effective procedure was designed for the purification of product HHD, which was isolated in up to 75 % yield, one the best result so far obtained. The CF reaction of HMF was instead explored in a H‐Cube® system. After a solvent screening at 100 °C and 50 bar H_2_, the process allowed the selective formation of the fully hydrogenation product BHMTHF in an ethyl acetate solution.

## Results and Discussion

### General: solvent and catalyst

The reactions of HMF are often reported using toxic non‐renewable solvents as tetrahydrofuran, 1,4‐dioxane, alcohols, and ionic liquids. In this work, with the aim of integrating the green chemistry principles in the development of sustainable synthetic protocols, both MP and CF experiments were carried out in solutions of water and ethyl acetate, respectively, which were chosen for their eco‐friendly nature. The use of water should be even more recommended in this case, in view of scaling up the reactions starting directly from HMF supplied as an aqueous stream from the biorefining of sugars. Details on the experimental procedures in all cases have been included in the Supporting Information.

Moreover, since carbon‐supported Ru is the most versatile catalyst for processing water‐soluble biosourced organic reactants,[^48^, ^49^] commercial 5 % Ru/C was used throughout this study to make the investigated procedure as easily accessible as possible. The catalyst for batch experiments was supplied by Sigma Aldrich (5 % Ru/C, lot MKBW5890 V) and was thoroughly characterized for its structural, morphological, and acid properties in recent papers by our group;[^38^, ^50^] a catalyst cartridge system (5 % Ru/C, CatCart®) supplied by ThalesNano Inc. was used for CF tests. All the reported reactions were run at least in duplicate to ensure reproducibility and, unless otherwise specified, conversions, selectivities, and isolated yields differed by less than 5 % from one test to another. It is worth to mention that some reports have investigated the catalytic activity of commercial Ru/C catalyst, revealing both the presence of partially oxidized ruthenium species and the synergistic effect of both Ru and RuO_2_ counterparts. X‐ray photoelectron spectroscopy (XPS) analyses have indicated that the surface of Ru/C catalyst is mainly composed of RuO_2_ (which could behave as a Lewis acid), and this species could be in‐situ reduced to metallic ruthenium (which is most likely responsible for the hydrogenolysis activity) under the reduction reaction conditions.^[51]^

### Batch hydrogenation/hydrogenolysis of HMF in aqueous solution

The catalytic hydrogenation of aqueous HMF was initially explored under conditions similar to those reported by Raspolli Galletti and co‐workers,^[36]^ except that a broader/different range of reaction temperature and pressure was investigated (Figure S1). Tests were carried out using an aqueous solution of HMF (0.2 m, 10 mL) in the presence of 5 % Ru/C (50 mg; Ru/HMF=1 wt %). This suspension was set to react under stirring in a stainless‐steel autoclave at different temperatures and pressures of H_2_ in the range of 40–100 °C and 5–50 bar, respectively, for 6 h.

**Scheme 2:**
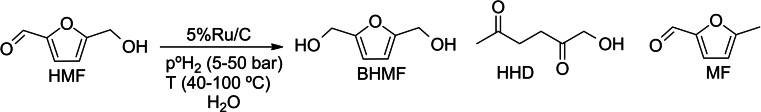
Hydrogenation/hydrogenolysis of HMF with 5 % Ru/C at 5–50 bar, 40–100 °C in water.

In comparison with other reports in the literature,^[36]^ where only the hydrogenation of HMF was observed, in our case (also considering the range of temperature and pressure employed herein), also hydrogenolysis products, mostly HHD, were detected (Scheme 2).

The structure of all products was identified by gas chromatography‐mass spectrometry (GC‐MS) and nuclear magnetic resonance (NMR) spectroscopy and confirmed by either independent syntheses or comparison to literature data (Figures S6–S19, Supporting Information).^[52]^ The conversion of HMF and products distribution were obtained by GC flame ionization detector (FID) analysis upon calibration with diethyleneglycol dimethylether as a standard (for details, see Figures S2–S4 in the Supporting Information).

In this work, given the interest for both the hydrogenation and the hydrogenolysis of HMF, the product selectivity was defined according to the following expression [Eq. 1]:

(1) $$S_{i}=\frac{n_{i}}{n_{HMF\;conv.}}\times 100$$

where *S*_i_ is the selectivity [%] for compound *i* (i. e., BMHF, HHD, etc.), *n*_i_ stands for the total moles of compound *i* (by GC calibration), and *n*_HMF conv._ is the converted moles of HMF (see calibration curves in Figures S20–S22) in all the processes where it is consumed (hydrogenation and hydrogenolysis). Moreover, the process carbon balance (%C_balance_) was determined as the difference of initial moles of HMF and the total molar amount of products [Eq. 2]:

(2) $${\%C}_{balance}=\frac{\sum n_{products}-n_{HMF\;initial}\;}{n_{HMF\;initial}}\times 100$$

The %C_balance_ is a relevant value for any reaction involving HMF to account for its limited thermal and chemical stability and its tendency to polymerize or degrade to humins and char.[^53^, ^54^, ^55^]

**Figure 1:**
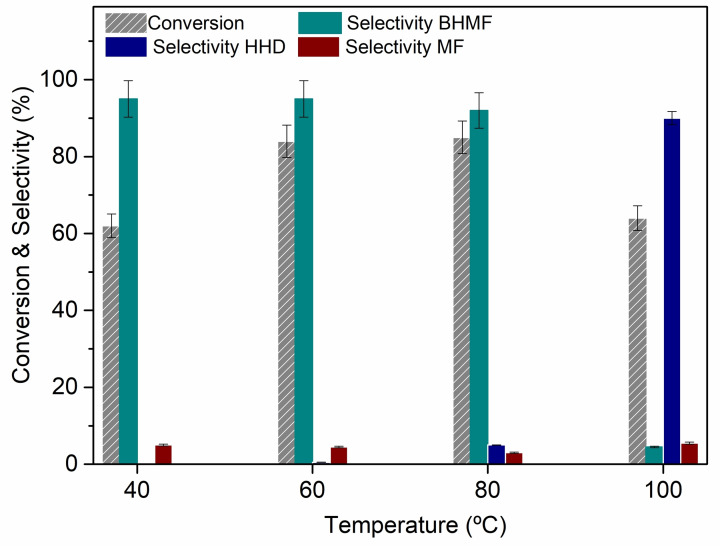
Effect of the temperature on the aqueous‐phase reaction of HMF. Conditions: *p*(H_2_)=30 bar, *t*=6 h, [HMF]=0.2 m (10 mL) in deionized water, 5 % Ru/C (50 mg).

Experiments proved that the products distribution was far more affected by the temperature than the pressure. The most representative results are summarized in Figure 1.

In the range of 40–80 °C, BHMF (this product was parallelly prepared for comparison according to Scheme S1) was consistently the major product, achieved with a selectivity of 92–95 %, at conversion of 63–85 %, respectively. Additional experiments reported in the Supporting Information (Figure S1) confirmed this trend: in general, the combined increase of *T* up to 80 °C and *p* to 30 bar mostly affected the conversion, which was enhanced to 94 %, while the BHMF selectivity was 95 %. Results were consistent with the effect of the temperature and the pressure on the hydrogenation kinetics and the H_2_ solubility in water. Benefits on conversion and selectivity, however, were limited above 30 bar. In all cases, the carbon balance was satisfactory, between 93 and 98 %.

The most striking evidence in Figure 1 was the change in the products distribution above 80 °C. By increasing the temperature from 80 to 100 °C, the selectivity dramatically shifted to 90 % towards HHD. Interesting was also the trend of HMF conversion that dropped from 85 % at 80 °C to 62 % despite the rise of *T* to 100 °C. Apparently, the HMF conversion was: (i) no longer improved by the temperature as soon as HHD started to form (6 %, 80 °C), and (ii) even disfavored when HHD became the predominant product at 100 °C.

**Scheme 3:**
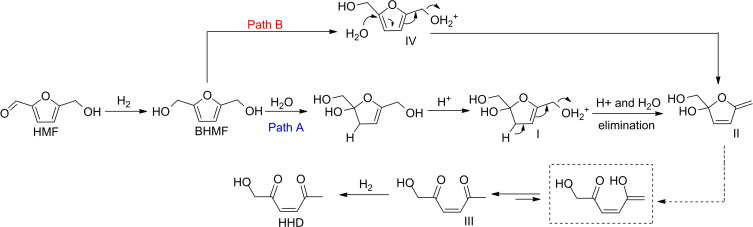
A plausible mechanism for the hydrolytic ring opening of HMF to HHD.

As mentioned in the introduction, studies on the synthesis of HHD from the hydrogenolysis of HMF have reported that the reaction occurs on the condition that a metal catalyst active for hydrogenation is assisted by an acidic environment.[^27^, ^28^, ^29^, ^30^, ^31^, ^32^] For example, in catalysts of Table 1 acidity is provided by either the catalyst support (e. g., Pd/Nb_2_O_5_), or the catalytic activation of H_2_ (e. g., Ir^III^+H_2_→Ir^III^−H+H^+^), or the external supply of CO_2_ for the in‐situ formation of carbonic acid. A mechanistic pathway for the formation of HHD was proposed accordingly (Scheme 3).

This formulation was originally suggested for the acid‐catalyzed conversion of HMF to levulinic acid, and then adapted to HHD.^[56]^ The initial step is the hydrogenation of HMF to BHMF. Thereafter, according to path A, a regioselective 2,3‐addition of water and OH‐protonation occur to provide the intermediate **I**. This in turn, undergoes a concerted elimination to provide the cyclic diene **II**. The same intermediate **II** is achieved by path B through an OH protonation of BHMF to yield **IV**, and a subsequent one‐step hydrolysis. Once **II** is formed, a ring‐opening reaction plausibly affords an enol species (dashed box, hypothesized compound) that tautomerizes to an α,β‐unsaturated carbonyl compound **III**. The final hydrogenation of **III** provides HHD. Details on the energetics of water addition/protonation to/of furan rings in acid water solutions have been described in a recent paper.^[57]^

Based on these considerations, the aqueous HMF solution used for the reactivity tests of Figure 1 was analyzed, and the corresponding pH was 3.2, consistent with the formation of HHD through Scheme 2. The acid contamination of HMF likely derived from the processing of sugars during the synthesis of commercial sample (in our case supplied by Merck).^[58]^ Other authors reported a similar evidence noticing that after removal of acid impurities (with a basic ion‐exchange resin), the selectivity of the hydrogenation of HMF to BHMTHF catalyzed by Ru‐black, was improved.^[34]^ In our case, an additional minor contribution to acidity in solution could come from the C support of the catalyst. Previous characterization tests^[38]^ indicated that different oxygen‐bearing groups were present on the surface of Ru/C used in this study. Temperature‐programmed desorption (TPD) analyses and Boehm titrations proved that functionalities including carboxylic, lactones, and phenol‐like ones imparted a total surface acidity of 140 μeq g^−1^.

Figure 1 also showed that the ring opening of HMF occurred to a very small extent (≈5 %) below 100 °C. A similar observation was reported for the hydrogenation/hydrogenolysis of HMF catalyzed by a Ni−Co‐Al mixed oxide system,^[22]^ where the formation of linear diols and polyols was noticed only at temperatures above 120 °C. These results were supported by a density functional theory (DFT) investigation on the competition between the hydrogenation and the ring opening of furan on Pd111.^[59]^ As well, some studies have been reported on the role of ruthenium for oxygenated furanics, indicating that DMF is a common reaction intermediate and therefore the reduction of the oxygenated substitutional groups is preferred to furan ring opening on Ru.^[60]^ The comparison of the activation energies and the kinetic analysis proved that at high temperatures, 1‐butanol (from ring opening) was the thermodynamically favored product, while at a low temperature, the ratio between the rate constants of furan hydrogenation (to THF) and furan ring aperture was around 6.^[59]^

**Figure 2:**
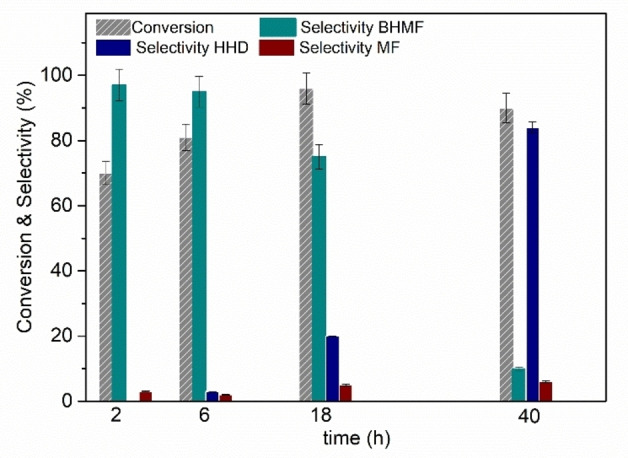
Effect of the reaction time on the batch reaction of HMF (autoclave). Conditions: 80 °C, *p*(H_2_)=30 bar, [HMF]=0.2 m (10 mL) in deionized water, 5 % Ru/C (50 mg).

This led us to assume that if BHMF and HHD were the kinetic and thermodynamic products of our experiments, respectively, a higher formation of HHD was likely at longer reaction times. Additional experiments carried out at 80 °C, under the conditions of Figure 1 but exploring an extended range of time from 2 to 40 h, corroborated this hypothesis. Results are reported in Figure 2.

The gradual increase of the reaction time from 2 to 18 h resulted not only in an enhancement of the conversion from 68 to 96 %, but also in the formation of an appreciable amount of HHD (18 %). By further prolonging the reaction to 40 h, HHD was obtained as a major product (84 %) at 90 % conversion. In comparison with results reported so far in the literature, this was one of the best achievements for the preparation of HHD. Mild conditions were possible by simply exploiting the presence of acid impurities in the HMF reagent: the energy supplied at a moderate temperature of 80 °C was enough to trigger the ring opening of HMF, thereby allowing the reaction mixture to equilibrate with time towards the thermodynamically favored product HHD. The trend of Figure 2 also corroborated the role of BHMF as an intermediate on the formation of HHD, in line with the mechanism of Scheme 2. This was confirmed by further tests carried out at 60 °C: after 40 h, even at such a low temperature, the reaction occurred with a significant ring opening of HMF, yielding 32 % HHD at complete conversion (compare Figure S5 in the Supporting Information).

Although less pronounced than in Figure 1, Figure 2 also showed a slight drop of the reaction conversion from 18 to 40 h, apparently correlated to the increased formation of HHD. The reasons for this behavior were not clear, but we hypothesized that the onset of the hydrolytic aperture of the furan species could adversely affect the adsorption of the same compounds over the catalyst surface during the hydrogenation step.

### Multiphase conditions for the hydrogenation/hydrogenolysis of HMF: products/catalyst separation

The cost of the catalyst in a liquid‐phase reaction may represent up to one third of the total cost of the process, implying that its loss (e. g., by leaching) can be critical, and its recovery and reuse are imperative. Moreover, the separation of C‐supported catalysts from organic/aqueous solutions is a well‐documented issue also in industry, where the sedimentation of fine powdered carbons with low particle sizes (the most active supports) in the reaction mixture is often a processing bottleneck, making filtration and reuse costly and time‐consuming.[^34^, ^61^, ^62^, ^63^]

Previous studies of our group have been aimed to cope with these problems via the design of MP systems able to confine the catalyst in a different phase from that where the reaction took place. MP systems comprised of mutually immiscible (aqueous/hydrocarbon/ionic liquid) phases proved successful for some representative hydrogenation processes including the conversion of levulinic acid into γ‐valerolactone, and sugars and sugar derivatives into the corresponding sugar alcohols.^[38]^ Under such conditions, the catalyst (Ru/C) was perfectly segregated in a hydrocarbon or an ionic liquid phase (where it was recycled), while the reagents and the products were consumed and formed in an aqueous solution, respectively. In this work, we were therefore prompted to explore whether the MP approach could be used also for the chemical upgrading of HMF. Experiments were carried out using a mixture of aq. HMF and 5 % Ru/C, to which isooctane as a hydrocarbon phase was added with and without the presence of an ionic liquid (IL). Based on our previous studies, trioctylmethyl phosphonium bis(trifluoromethane)sulfonimide ([P_8881_][NTf_2_]) was chosen as the ionic liquid for its thermal stability (relevant to design reactions at *T*≥100 °C) and the lipophilic nature of the [NTf_2_] anion that reduced the IL/water miscibility. [P_8881_][NTf_2_] was synthesized according to a procedure reported by us (see Scheme S2a,b, Supporting Information).^[63]^

Starting from conditions of Figure 2, an extensive screening of the relative proportions of the MP components was explored by varying the HMF/catalyst molar ratio (*W*) from 100 : 1 (aq. HMF: 0.2 m, 10 mL; 5 % Ru/C: 50 mg) to 50 : 1 (HMF: 0.05 m, 10 mL; 5 % Ru/C: 25 mg), and the quantity (*Q*) of the IL between 355 to 657 mg corresponding to around 0.6 and 1 mmol, respectively. The volume of hydrocarbon phase was kept constant to 5 mL.

In the presence of isooctane alone, the reaction outcome did not significantly change compared to the experiments of Figure 2 in aqueous HMF solutions (see Table S1 in the Supporting Information). From visual inspection, however, it was clear that the desired segregation of Ru/C in the hydrocarbon phase did not occur. It was hypothesized that a strong adsorption also involving H‐bonding interactions of the water‐soluble reactant and products with the surface carboxylic and phenolic groups on the carbonaceous support of the catalyst retained a portion of Ru/C suspended in the aqueous phase.

**Figure 3:**
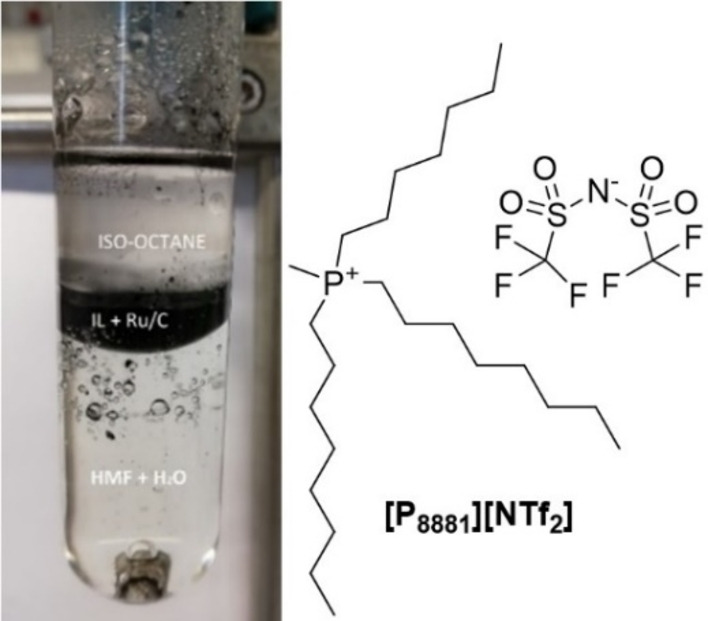
MP‐mixture comprised of aqueous solution ([HMF]=0.05 m, 10 mL), isooctane (5 mL), Ru/C (25 mg), and [P_8881_][NTf_2_] as an ionic liquid (650 mg).

By contrast, in the presence of [P_8881_][NTf_2_], the MP system proved effective for the catalyst segregation. Optimized values were found at *W*=50 and *Q*=657 mg (Figure 3).

**Figure 4:**
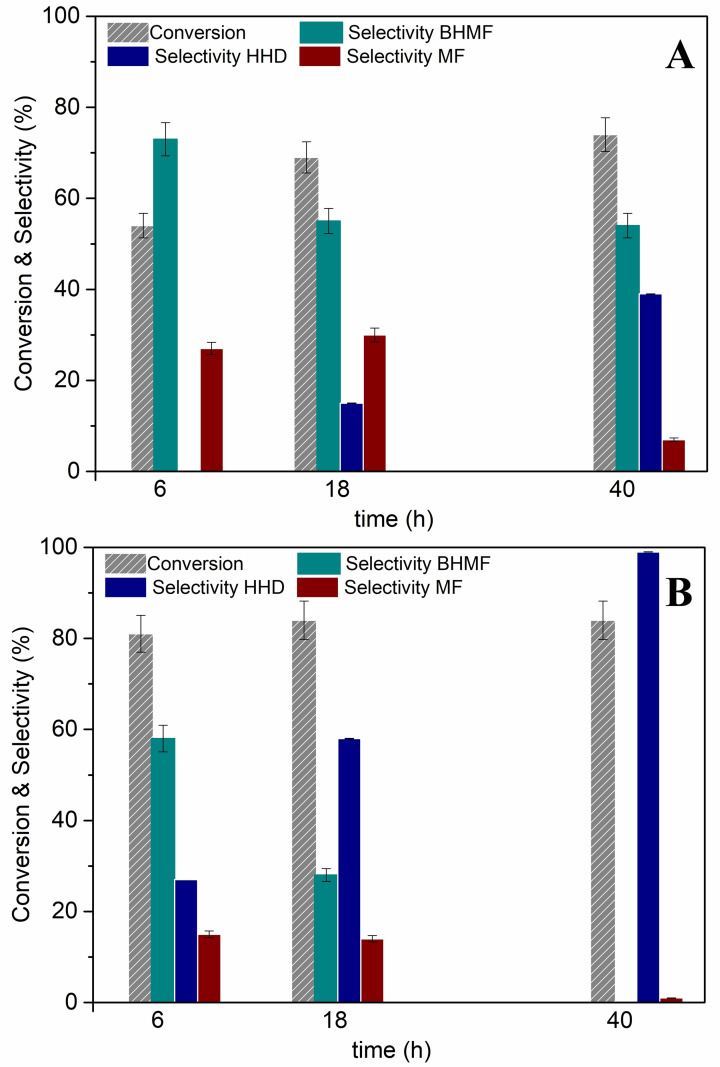
Effects of temperature and time for the multiphase reactions of HMF. Conditions: HMF (0.05 m, 10 mL), isooctane (5 mL), Ru/C (25 mg), and [P_8881_][NTf_2_] (650 mg), *p*(H_2_)=50 bar. (A): 60 °C; (B): 80 °C.

The substrate (HMF) and the catalyst were perfectly confined in the aqueous and IL phases, respectively. Although the role of isooctane was inconsistent as a solvent for the chemical species, it was necessary, however, to obtain phase separation and catalyst segregation. Under the conditions of Figure 3, experiments were carried out at 60 and 80 °C for 6, 18, and 40 h in each case. The pressure of H_2_ was set to 50 bar to favor the gas solubility in the involved phases, particularly in the viscous IL. Results are reported in Figure 4.

Although conditions were not strictly comparable to those used in Figures 1 and 2, MP tests proceeded with a general decrease of the conversion and, most importantly, an alteration of the products distribution compared to reactions carried out in water alone. At 60 °C, the HMF conversion was enhanced from 53 to 77 % when the reaction time was increased from 6 to 40 h. Products from hydrogenolysis and ring opening reactions were obtained in relatively high amounts and variable with time: the selectivity towards MF and HHD was 28 and 0 % after 6 h,^[64]^ and 7 and 39 % after 40 h, respectively. At 80 °C, the conversion reached a plateau at around 85 % after 18 h, and it did not improve further by prolonging the reaction to 40 h. HHD became the predominant (58 %) and then the sole product (99 %) after 18 and 40 h, respectively.

Thanks to the combination of the acidic aqueous environment and the MP system mediated by the ionic liquid, the hydrolytic ring opening of HMF to HHD was far more selective than that achieved in the aqueous (single‐phase) solution (Figure 2).

Several previous investigations clearly demonstrated that the performance of different active metals (Pd/C, Pt/C, Ru/C, and Raney‐Ni) were significantly affected by their confinement in ionic liquid media.^[65]^ The occurrence of polar interactions between the catalyst (mostly through its support) and the IL was claimed responsible for the embodiment of the metal in a dense‐viscous environment. The IL acted concurrently as a catalyst‐philic phase and as an interfacial boundary layer, which mediated the migration (adsorption/desorption) of the liquid/gaseous reagents and products to and from the catalyst, respectively, therefore impacting both conversion and products distribution of MP reactions. This could also imply changes in the reaction mechanism as was suggested by the presence of MF under the conditions of Figure 4. MF rose up to a significant amount (25–28 and 12–13 % at 60 and 80 °C, respectively) and then it dropped until its almost total disappearance. This intermediate‐like behavior could be consistent with an involvement of MF in the formation of HHD through a pathway different than that of Scheme 2.

Whatever the role of the ionic liquid, the synthetic value of the finding of Figure 4 prompted us to further investigate the effect of the temperature by designing additional experiments at 100 and 120 °C, respectively. Both the MP composition (aqueous HMF: 0.05 m, 10 mL; isooctane: 5 mL; [P_8881_][NTf_2_]: 650 mg) and the H_2_ pressure (50 bar) were kept unaltered. Results are summarized in Table 2.

**Table 2:** MP reaction of HMF in water/isooctane system/IL multiphase conditions.^[a]^

Entry	T [°C]	t [h]	Cat. [mg]	Conv. [%]	Product sel. [%]	Carbon balance [%]	
					HHD	MF	
1	120	18	25	>99	99	1	94
2	100	18	25	83	99	1	96
3	100	18	50	>99	99	1	95

[a] Reactions were carried out using a multiphase system comprised of an aqueous solution of HMF (0.05 m, 10 mL), isooctane (5 mL), and [P_8881_][NTf_2_] (650 mg) under 50 bar of H_2_.

The rise of *T* to 120 °C brought about the desired enhancement of the conversion, with no side‐effects on the selectivity. The reaction was quantitative and proceeded with the exclusive formation of HHD after 18 h (Table 2, entry 1). Under such conditions, however, the catalyst did not appear neatly confined in the IL‐phase at the end of the experiment. This behavior was associated to a partial degradation of the IL due to the combined (and prolonged) action of the temperature and the acid environment.^[66]^

However, further optimization tests carried out at 100 °C proved successful to achieve complete catalyst segregation according to Figure 3, full conversion, and 99 % selectivity to HHD after 18 h by employing 50 mg of catalyst (entry 3). The corresponding carbon balance was 95 %. The isolation of HHD was then addressed. As mentioned in the Introduction, this is a tricky step. A procedure was identified by simplifying the method described by de Vries and co‐workers:^[32]^ (i) at the end of the reaction, the aqueous phase containing HHD was separated, and the solvent (water) evaporated; (ii) the oily residue was dissolved in dichloromethane (2 mL), dried over Na_2_SO_4_, filtered, and added with *n*‐hexane (10 mL); (iii) the biphasic solution was allowed to stand for 24 h at −10 °C. HHD finally separated as a yellow solid in a 75 % yield (GC purity >99 %). Although the use of harmful solvents could not be avoided, the overall result was superior to the best one reported so far using a tailored homogeneous iridium catalyst (Table 1, isolated yield: 69 %). Moreover, thanks to the MP system, the protocol did not require any flash column chromatography (FCC) purification step otherwise necessary to remove the homogenous Ir‐complex. Encouraged by this excellent outcome, the study was then focused on the potential of MP conditions for the recycling of the catalyst.

### Recycle and leaching tests of Ru/C in the multiphase system

**Figure 5:**
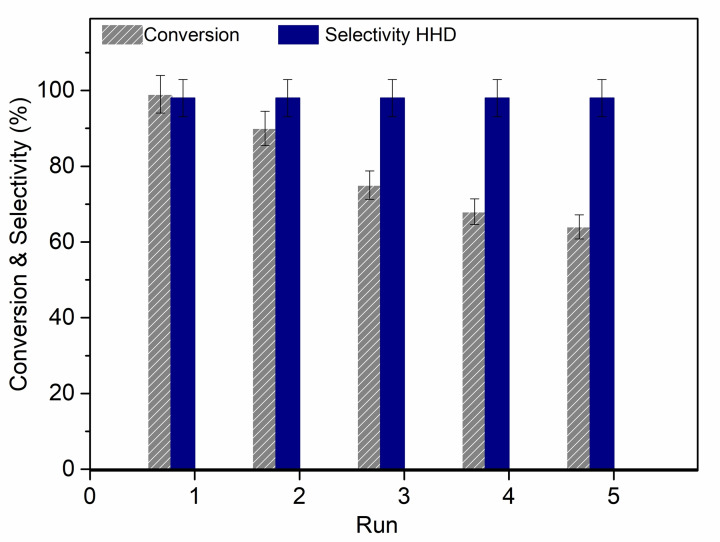
Recycling of Ru/C in five subsequent runs in the conversion of HMF to HHD. Conditions: [HMF]=0.05 m in 10 mL of deionized water, Ru/C=50 mg, [P_8881_][NTf_2_]=650 mg, isooctane=5 mL, *T*=100 °C, *p*(H_2_)=50 bar, *t*=18 h.

Tests for the recycling and reuse of the catalyst were designed under the conditions of entry 3 of Table 2 ([HMF]=0.05 m, 50 mg Ru/C, 50 bar, 100 °C, 18 h). Once a first reaction was complete (run 1), the aqueous solution was removed, and the remaining phases comprised of the IL (with the confined catalyst) and isooctane were washed with milli‐Q water (2×10 mL) and added with a fresh aqueous HMF solution (0.05 m, 10 mL) to run a second reaction. The whole sequence was repeated up to five subsequent runs. The results are reported in Figure 5.

Ru/C appeared entirely segregated in the IL‐phase at the end of each test. After the first quantitative reaction (run 1), the conversion of HMF progressively decreased to 64 %, thereby suggesting that the catalyst (partially) deactivated during the recycling tests. The selectivity towards HHD however, remained steady at 99 %.

Studies on the reasons responsible for deactivation ruled out metal leaching: inductively coupled plasma (ICP) analyses of the aqueous phase recovered at the end of run 5 proved that the Ru leaching was negligible (<0.01 %; analytical details are given in Table 3 and Figure S23). Deactivation was, instead, most plausibly due to the presence of poisoning organic moieties, which were co‐adsorbed on the catalyst surface. This was confirmed once the catalyst (Ru/C) recovered after recycling tests, was thoroughly washed with AcOEt (10 mL), treated at 300 °C in H_2_ flow (2 mL min^−1^) for 5 h, and reused under the conditions of Table 2, entry 3. The catalytic activity was completely restored with 99 % selectivity towards HHD at complete conversion.

**Table 3:** ICP analysis of spent aqueous phases recovered after multiphase reactions of HMF.

Entry	Reaction parameters	Ru [ppm]	Ru[a] [wt %]
1	Ru/C=50 mg, 50 bar H2, 100 °C, 18 h	0.02	0.01

[a] Metal dissolved in water with respect to the amount of Ru in the catalyst used for the reactivity tests.

### Effect of acidity

**Figure 6:**
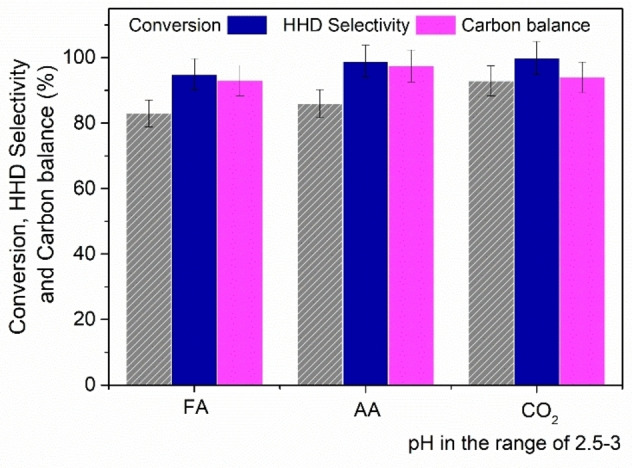
Effects of acidity on the synthesis of HHD at 100 °C. Conditions: t=18 h, [HMF]=0.05 m (10 mL) in deionized water, 5 % Ru/C (25 mg), 650 mg TOMP‐NTf_2_, isooctane 5 mL, 50 bar H_2_. When CO_2_ was used, 30 bar were loaded after the pressurization with H_2_ (50 bar). FA: formic acid, AA: acetic acid.

As mentioned above, the conversion of HMF to HHD took advantage of the residual acidity (acid impurities) of the starting reactant. This aspect was further inspected by purifying the commercial sample and then controlling the pH of the reaction environment by an external acid supply. Accordingly, commercial HMF used in this work was subjected to FCC (on silica; Et_2_O: 100 %). The residual solid gave almost neutral solutions (pH=6.5 at 0.05 m), to which formic acid (FA) or acetic acid (AA) were added to adjust the pH at 2.5. MP tests were then carried out under the conditions of Table 2, entry 2. An additional reaction was run by adding CO_2_ (30 bar) without any organic acid. In this case, pH was around 3.0 due to carbonic acid.^[67]^ Results are reported in Figure 6.

The reaction conversion (82–88 %), HHD selectivity (95–98 %) and carbon balance (92–97 %) achieved by either the presence of FA, AA, or H_2_CO_3_, substantially matched the values of Table 2 (entry 2), thereby fully supporting the role of (controlled) acidity on the investigated reaction.

### Hydrogenation of HMF in continuous‐flow mode

Furthermore, considering the advantages of CF operational conditions for scale‐up applications, additional experiments were performed using a CF hydrogenation reactor, namely the H‐Cube® apparatus.

#### Influence of flow rate

**Scheme 4:**
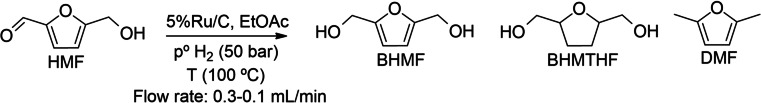
CF‐hydrogenation/hydrogenolysis of HMF at 100 °C and 50 bar in EtOAc as a solvent.

The CF hydrogenation/hydrogenolysis of HMF was explored under conditions as close as possible to those used in the batch mode in order to compare the two methodologies. Initial tests were run at 100 °C, *p*(H_2_)=50 bar, using a solution of HMF in ethyl acetate (EtOAc; 0.05 m) (see Scheme 4). This reaction was carried out at two flow rates of 0.1 and 0.3 mL min^−1^, respectively, using a catalytic bed of 5 % Ru/C (0.3 g). The selection of CF conditions was based on a previous work from our research group on the hydrogenation of HMF derivatives as 5‐methoxymethylfurfural (MMF).^[68]^

**Figure 7:**
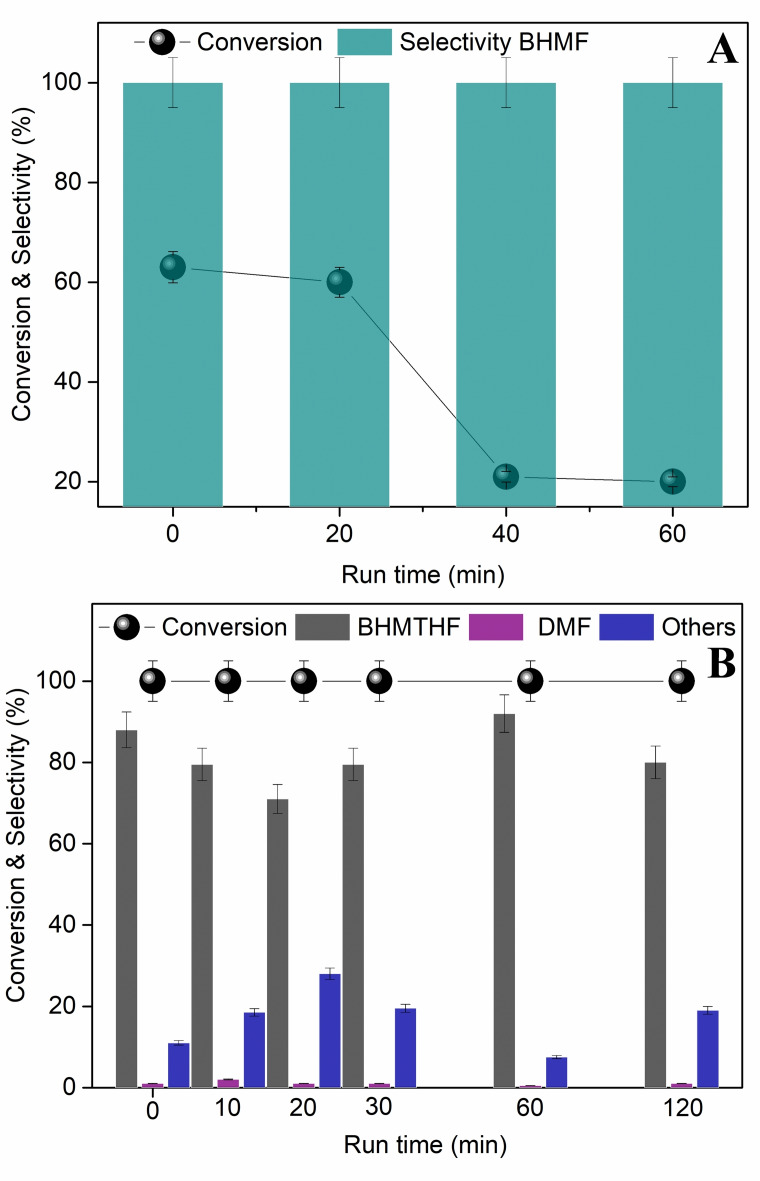
HMF hydrogenation in continuous‐flow mode. Conditions: *p*(H_2_)=50 bar, *T*=100 °C, HMF in EtOAc (0.05 m), Ru/C (0.3 g) in a CatCart® capsule. (A) Flow rate=0.3 mL min^−1^; (B) flow rate=0.1 mL min^−1^.

Figure 7 reports the results by showing the effect of the time‐on‐stream on the conversion and selectivity to BHMTHF, BHMF, and DMF. At *F*=0.3 mL min^−1^, a rapid decrease of conversion with time was observed, from 65 % at *t*_0_ (when the first sample was collected at the outlet of H‐Cube®, typically after ≈18 min from the start of flowing the mixture in the reactor), to less than 20 % at *t*_60_, after 1 h of reaction. By contrast, the selectivity to BHMF was 100 % throughout the experiment (Figure 7A).

By reducing the flow rate to 0.1 mL min^−1^, a steady quantitative conversion was obtained for 120 min. However, as a result of the higher contact time, the fully hydrogenated product BHMTHF became the major derivative (75–90 %; Figure 7B), and the onset of hydrogenolysis reactions of HMF brought about the formation of DMF (5 %). A non‐negligible formation of unidentified side‐compounds (“others”, up to 25 %) was also noticed. In this case, however, the products distribution showed an oscillating trend and therefore a poor fidelity of the CF experiment.

#### Influence of time‐on‐stream and HMF concentration

**Figure 8:**
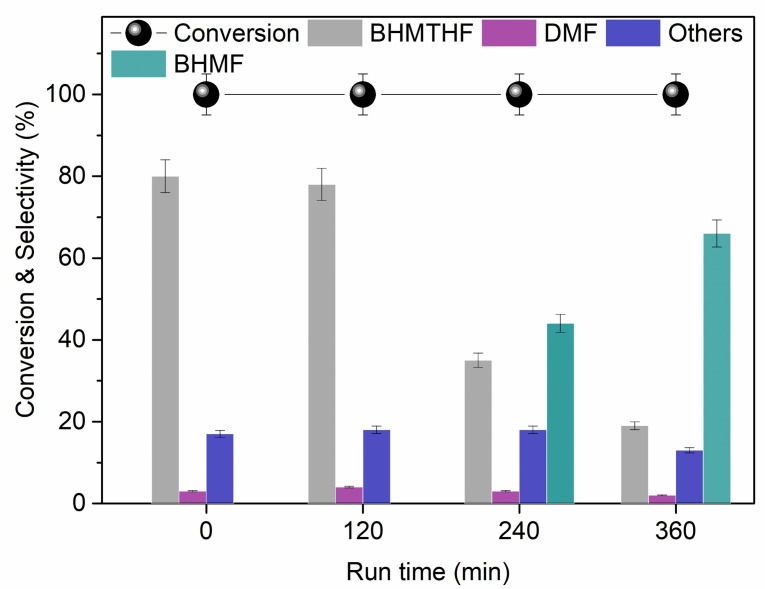
HMF hydrogenation in continuous‐flow mode. Conditions: *p*(H_2_)=50 bar, *T*=100 °C, HMF in EtOAc (0.05 m), Ru/C (0.3 g) in a CatCart® capsule, flow rate=0.1 mL min^−1^.

**Scheme 5:**
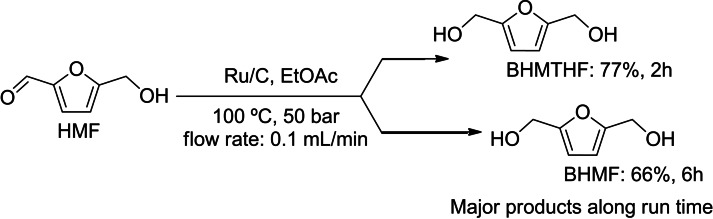
Product distribution of HMF hydrogenation in the continuous‐flow experiment using *F*=0.1 mL min^−1^, at 2 and 6 h.

With the aim of exploring the effect of time‐on‐stream, prolonged CF experiments were carried out for 6 h under the conditions of Figure 7B (*F*=0.1 mL min^−1^). The outcome of these reactions is displayed in Figure 8. Although the conversion continued to remain quantitative, the products distribution completely changed after 6 h compared to the results previously achieved at 2 h. Particularly, the less hydrogenated derivative BHMF (66 %; from the reduction of HMF carbonyl function) was observed with the consequent decrease of BHMTHF selectivity, which dropped to 18 % (Scheme 5). The relative amount of side‐products indicated as “others” did not vary appreciably with time, oscillating in the range of 12–18 %. This trend highlighted a diminishing of the hydrogenation performance of the catalyst, thereby indicating that saturation or deactivation phenomena were plausible in the long run.

Experiments carried out by halving the HMF concentration (0.025 m) and varying the flow rate between 0.1 and 0.3 mL min^−1^ led to the same conclusions. Results are illustrated in Figure S24. At *F*=0.1 mL min^−1^, the progress of the reaction was comparable to that previously observed at [HMF]=0.05 m. The fully hydrogenated product, BHMTHF, was the major product up to 79 % for the first 3 h. Then, the formation of BHMF was observed, which gradually increased to 35 % after 6 h. The conversion was quantitative throughout the test. When the flow rate was increased to 0.3 mL min^−1^, not only did the conversion drop rapidly from 100 to 65 % after 120 min, but in the same interval of time, the selectivity changed in favor of the less‐hydrogenated BHMF, which became the predominant product (90 %). Again, results suggested a deactivation/saturation of the catalyst with time.

#### Influence of solvent

THF and ethanol were considered as alternative solvents for the CF reaction of HMF. Tests were carried out under conditions that led to complete conversion for a relatively long reaction time (up to 6 h) and a moderate formation of by‐products, in accordance with previous experiments. Accordingly, reactions were run for 6 h, at 100 °C under 50 bar of pressure and employing a flow rate of 0.1 mL min^−1^. Solutions of HMF (0.05 m) in THF or EtOH, respectively, were used instead of ethyl acetate.

In the presence of THF as the solvent, the formation of BHMTHF gradually increased, with a selectivity ranging from 57 % at *t*_0_ to a steady value of around 80 % at *t*_360_. The conversion, however, significantly decreased from 100 to 48 % in the same time interval (Figure S25).

The use of ethanol as the solvent resembled that of ethyl acetate, although some differences were appreciated. The conversion of HMF was always quantitative during the explored time interval. BHMTHF was the initial major product (55–58 %), though its formation was accompanied by the presence of relative high amounts of side‐products (“others”: ≈35 %). After the first 3 h, both BHMTHF and “others” decreased in favor of the less‐hydrogenated derivative BHMF, whose selectivity raised up to 60 % at 6 h (Figure S26). Notably, under these conditions, BHMF underwent a partial etherification with ethanol yielding 2‐(hydroxymethyl)‐5‐(ethoxymethyl)furan (13 %), which was identified among products in the reaction mixture.

The formation of partially hydrogenated products, particularly BHMF, was preferred at the low residence time (2.6 min) achieved with a flow rate of 0.3 mL min^−1^. These conditions also favored side reactions of hydrogenolysis yielding comparably high amounts of undesired compounds (“others”). As expected, when the residence time was increased (8 min at 0.1 mL min^−1^), the fully hydrogenated product, BHMTHF, was predominant.

The solvent also induced significantly different reaction outcomes with ethyl acetate and ethanol that allowed a steady conversion, in comparison with THF, but a faster change of selectivity as well. Varying the HMF concentration in the range of 0.025–0.05 m did not result in significant improvements in the reaction outcome, except for increasing (at 0.05 m) or decreasing (at 0.025 m) the time at which the products distribution changed in the direction of favoring partially hydrogenated derivatives (Figures S24 and S25). Indeed, this variation of the selectivity was the major issue of the CF reaction, consistent with a plausible loss of hydrogenation activity of the catalyst, most likely related to the degradation of HMF or its derivatives and the formation of carbonaceous deposits on the catalyst surface.

## Conclusions

The poor stability of 5‐hydroxymethylfurfural (HMF), plausibly responsible for the variable purity of commercial samples, together with the presence of acid impurities are major issues when studying the reactivity of this compound. Moreover, also hydrogenation derivatives of HMF prove sensitive to chemical modifications with time, and their isolation is often complicated. Notwithstanding these aspects, the herein developed investigation has highlighted some salient features of the reaction of aqueous HMF under H_2_ pressure and in the presence of commercial 5 % Ru/C as a catalyst. It has been demonstrated that the selective formation of two different products, namely 2,5‐bis(hydroxymethyl)furan (BHMF) and 1‐hydroxyhexane‐2,5‐dione (HHD) can be tuned by the reaction conditions, and interestingly, for the first time, the synthesis of HHD has been described in an ionic liquid‐assisted multiphase system. The reaction temperature resulted to be the most critical parameter to steer the conversion/selectivity, while the pressure of H_2_ displayed a lower impact (Figure S1). In line with the many reported papers on the subject, BHMF has been obtained as an almost exclusive product (95 %) from the partial hydrogenation of aqueous HMF at 40–80 °C. However, it has been noticed that the increase of temperature to 100–120 °C favored the hydrolytic ring opening of HMF, yielding HHD as the sole derivative at complete conversion. A further analysis of the reaction system has proved that the selectivity shift from BHMF to HHD was likely due to the combined effects of the temperature and an acidic environment. Such result, together with the possibility to in‐situ recycle the catalyst, prompted us to transfer the process from a single aqueous solution to a multiphase system comprised of three immiscible phase including water, isooctane, and an ionic liquid, trioctylmethylphosphonium bis(trifluoromethane)sulfonimide [P_8881_][NTf_2_]. The multiphase system resulted to be an outstanding approach, not only to achieve high conversion and selectivity similar to that observed in the aqueous solution, but to allow the catalyst/product separation, with Ru/C and the product (HHD) perfectly segregated in the IL and water, respectively. At the present stage, however, recycling of the catalyst showed a partial drop of its performance after five reuse cycles, most likely due to the adsorption of organic moieties on the catalyst surface. The hydrogenation/hydrogenolysis of HMF was also studied in a continuous‐flow mode. The achieved results revealed the formation of BHMF and 2,5‐bis(hydroxymethyl)tetrahydrofuran (BHMTHF) as main products. Several factors influencing the selectivity of the process were investigated, including flow rate, solvent, and time. In particular, time on‐stream displayed a critical influence on the products distribution, suggesting that the catalytic bed based on Ru/C undergoes deactivation under the investigated conditions. Undoubtedly, HMF valorization is, at the same time, a promising and challenging goal, which still requires a lot of efforts from the scientific community. In particular, this contribution has aimed to open new possibilities for the use of multiphase and continuous‐flow systems for HMF upgrading.

## Experimental Section

### Materials and instruments

All chemicals were purchased from Sigma Aldrich and used without any further purifications. Water was milli‐Q grade. H_2_ and N_2_ gases were purchased from SIAD, Italy. Reactions of HMF were performed in a jacketed stainless‐steel autoclave (150 mL internal volume) equipped with a manometer and two needle valves for gas admission and purging, respectively. The reactor was kept at the desired temperature by an oil circulation external thermostat.

Syntheses of BHMF, DFF, and [P_8881_][NTf_2_] were carried out as described later in this section. ^1^H NMR spectra were recorded at 400 and 300 MHz, ^13^C NMR spectra were recorded at 100 MHz, and ^31^P NMR spectra were recorded at 161 MHz. Chemical shifts are reported downfield from tetramethyl silane (TMS), and CDCl_3_ or DMSO‐d6 were used as the solvents.

Pd/C and Ru/C CatCart® were purchased from ThalesNano.93. CF hydrogenation reactions of HMF were carried out in a ThalesNano H‐Cube® Mini Plus able to operate up to 100 °C and 100 bar, with a flow rate between 0.1 and 3 mL min^−1^, and a capacity for hydrogenation generation of 25–30 mL min^−1^. Analyses of the reaction mixtures were performed as follows: (i) GC‐MS equipped by an electron ionization (EI) source at 70 eV using a HP‐5 MS ultra‐inert column (*L*=30 m, Ø=0.25 mm, film=0.25 μm), with the following method: 1 min at 50 °C, 10 °C min^−1^, 10 min at 230 °C. (ii) GC‐FID using two different columns, a HP‐5 capillary column (*L*=30 m, Ø=0.32 mm, film=0.25 μm) with the following method: 2 min at 105 °C, 20 °C min^−1^, 3 min at 150 °C, 25 °C min^−1^, 3 min at 210 °C, and a Restek Rt®‐yDEXsa column (*L*=30 m, Ø=0.25 mm, film=0.25 μm) with the following method: 3 min at 60 °C, 20 °C min^−1^, 10 min at 170 °C.

### Hydrogenation/hydrogenolysis of HMF in aqueous solution

Tests were carried out using an aqueous solution of HMF (0.2 m, 10 mL) in the presence of 5 % Ru/C (50 mg; Ru/HMF=1 wt %). This suspension was set to react under stirring in a stainless‐steel autoclave at different temperatures and pressures of H_2_ in the range of 60–80 °C and 5–50 bar, respectively, for 6 h. The conversion of HMF and products selectivity were obtained by GC‐FID analysis upon calibration with diethyleneglycol dimethylether as a standard (Figures S2–S4).

### Reaction of aqueous HMF at 60 °C

Experiments were carried out under the same conditions of Figure S1 except for the temperature, which was decreased to 60 °C. A mixture of HMF (0.2 m, 10 mL) in deionized water and 5 % Ru/C (50 mg) was set to react under *p*(H_2_)=30 bar, by exploring an extended range of time from 2 to 40 h.

### Multiphase reaction of HMF in aqueous/isooctane biphasic system

Experiments were carried out under the conditions of Figure S1 using a mixture of aqueous HMF (0.2 m, 10 mL) and 5 % Ru/C (50 mg). The MP system was established by adding isooctane (5 mL).

### Synthesis of BHMF

BHMF was synthesized using NaBH_4_ as a stoichiometric reductant (Scheme S1). This preparation was aimed to have a standard product available and confirm its presence during the hydrogenation of HMF investigated in this study. Accordingly, two aqueous solutions were prepared by first dissolving HMF (1.26 g, 10 mmol) in milli‐Q water (20 mL), and then, NaBH_4_ (0.38 g, 10 mmol) in milli‐Q water (4 mL). Aqueous NaBH_4_ was added dropwise to the HMF solution under vigorous stirring. The exothermicity of the reaction was controlled by keeping the mixture in a water bath at room temperature for 2 h. BHMF was extracted with ethyl acetate (4×15 mL). The isolated product was analyzed by GC‐MS and NMR spectroscopy.

### General procedure for hydrogenation of HMF in multiphasic batch system

**Reactions carried out in the presence of HMF aqueous solutions alone**: Commercial 5 % Ru/C (50 or 25 mg) was introduced to a 25 mL tubular reactor of borosilicate glass (Pyrex) equipped with a magnetic stirrer. Thereafter, an aqueous solution (10 mL) of HMF (0.2 or 0.05 m) was added to the reactor, which was closed with a holed glass cap (for pressure equilibration) and placed in the above‐described jacketed steel autoclave. The system was then pressurized with gaseous H_2_ (in the range of 5–50 bar), heated at the desired temperature by oil circulation (40–100 °C), and the mixture was stirred at 1300 rpm. At the end of the experiment (2–40 h), the autoclave was cooled to room temperature by a water bath and slowly purged. An aliquot (0.5 mL) of the aqueous solution was withdrawn, filtered on Celite®545 to stop undesired, if any, Ru/C particles and added to an aqueous solution of diethylene glycol dimethyl ether (0.5 mL, 0.01 m) used as an external standard. The mixture was analyzed by GC‐FID to determine the conversion of HMF and the selectivity toward the products. The conversion of HMF was determined by calibration with standard aqueous solutions. The structure of the reaction products was assigned by GC‐MS and by comparison to literature data. In the case of BHMF, the structure was further validated by comparison with the independently synthesized product. In the case of HHD, the product was isolated as described in the paper, and identified by NMR spectroscopy.

**Reactions carried out under multiphase conditions**: The procedure above described for HMF aqueous solutions alone was used also for multiphase reactions. Leaving other conditions unaltered, after the loading of aqueous HMF and the catalyst, the glass reactor was added with either isooctane (5 mL) or a mixture of isooctane (5 mL) and [P_8881_][NTf_2_] (0.65 g, 1 mmol) as an ionic liquid.

**Catalyst recycle procedure**: At the end of a typical multiphase experiment, the aqueous phase was siphoned out of the glass reactor using a needle, under a moderate N_2_ pressure. Fresh milli‐Q water (10 mL) was added to wash the residual phases (Ru/C, isooctane, and IL). The system was stirred for 1 h, after which water was replaced (by the siphoning method) with a fresh aqueous HMF solution (10 mL). The multiphase mixture was then set to react according to the above‐described procedure.

**Leaching tests**: Measures were performed using an Agilent 4210 MP‐AES microwave plasma atomic emission spectroscopy. The primary ionization excitation wavelength signal centered at *λ*=372.8 nm was selected to detect Ru. A standard solution of RuCl_3_ (1000 mg L^−1^, HCl 10 %) was diluted to prepare six aqueous solutions containing 0.05, 0.1, 0.25, 0.5, 1, and 2.5 ppm of Ru; each one of these was analyzed by repeating the measurement five times. Thereafter, the aqueous sample collected after recycling tests (Figure 5) from water/IL/isooctane multiphase experiments was analyzed. The detected amount of Ru was 0.01 ppm. An aqueous solution of HMF (0.05 m) was also analyzed as a blank.

### Hydrogenation of HMF in continuous‐flow mode

In a typical procedure, H‐Cube® Mini Plus, equipped with a 70 mm CatCart® (5 % Ru/C: 0.3 g), was flushed with methanol (0.1 mL min^−1^; 30 min) and then with the solvent used for the reactions (EtOAc, THF, EtOH; 0.1 mL min^−1^ for 30 min at room temperature). After conditioning, a solution of HMF (0.05–0.025 m), with cyclohexane as an internal standard, was pumped through the catalytic bed, and temperature, pressure, and flow rate were set up to the desired conditions. The time corresponding to the dead volume of the instrument was waited for the system to stabilize. Then, the sampling began. Samples were analyzed by GC‐FID. The main reaction products were identified by GC‐MS, and their structures were confirmed by comparison to authentic standards (BHMF, DMF, BHMTHF). Other products were not identified and were indicated as “others”. After the reaction was complete, both system and catalyst were washed thoroughly with the reaction solvent and methanol. The CatCart® containing the catalyst was then dried in a stove at 80 °C overnight before its further use.

## Conflict of interest

The authors declare no conflict of interest.

1

## Supporting information

As a service to our authors and readers, this journal provides supporting information supplied by the authors. Such materials are peer reviewed and may be re‐organized for online delivery, but are not copy‐edited or typeset. Technical support issues arising from supporting information (other than missing files) should be addressed to the authors.

Supporting Information

## Data Availability

Research data are not shared.
