# Supplementary material for: Tuning the Selectivity of the Hydrogenation/Hydrogenolysis of 5‐Hydroxymethylfurfural under Batch Multiphase and Continuous‐Flow Conditions
Source: ChemSusChem. 2022 Jun 28;15(13):e202200503. doi: 10.1002/cssc.202200503 (PMC9400871; doi:10.1002/cssc.202200503)
Supplement: Supplementary file 1 — Supporting Information [file CSSC-15-0-s001.pdf]

ChemSusChem

Supporting Information

Tuning the Selectivity of the Hydrogenation/Hydrogenolysis of 5-Hydroxymethylfurfural under Batch Multiphase and Continuous-Flow Conditions

Daily Rodríguez-Padrón, Alvise Perosa, Lilia Longo, Rafael Luque,* and Maurizio Selva*This publication is part of a collection of invited contributions focusing on “Green Conversion of HMF”. Please visit to view all contributions.© 2022 The Authors. ChemSusChem published by Wiley-VCH GmbH. This is an open access article under the terms of the Creative Commons Attribution License, which permits use, distribution and reproduction in any medium, provided the original work is properly cited.

The hydrogenation/hydrogenolysis of HMF in aqueous solution.

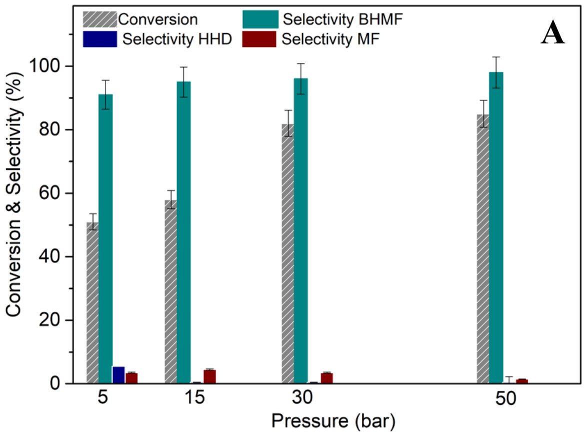

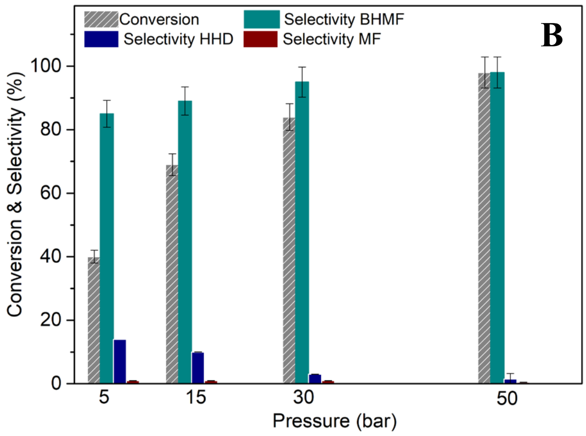

Figure S1. Comparative effects of T and H_2 pressure on the hydrogenation of HMF performed in a batch reactor (autoclave). A: T = 60 °C, pressure from 5 to 50 bar. B: T = 80 °C, pressure from 5 to 50 bar. Other conditions: t = 6 h, [HMF] = 0.2 M (10 mL) in deionized water, 5% Ru/C (50 mg). Conversion of HMF and products selectivity were obtained by GC-FID analysis.

Figure S1 shows the trend of conversion and products distribution as a function of T and p, after 6 hours. At 60 °C, increasing the pressure of H_2 from 5 to 50 bar, resulted in a significant increase of the conversion from 51% to a plateau value of 84%, and in a slight, but appreciable improvement of the BHMF selectivity from 92% to 97% (Figure S1 A). The combined increase of T to 80 °C and p in the interval of 15-50 bar, had similar consequences; it mostly affected the conversion which was enhanced from 68% up to 94%, while the BHMF selectivity ranged from 88% to 94% (Figure S1 B). Unexpectedly, compared to the process at 60 °C, the reaction carried out at 80 °C and 5 bar proceeded with a lower conversion and BHMF selectivity (41% and 84%, respectively), and a concurrent not negligible formation of HHD (12%) (Figure S1 B). Even if an explanation of this behavior would have required a deeper analysis, we hypothesized that the combined effect of the higher T and the modest availability of H_2 (in the aqueous reactant

solution) at 5 bar, plausibly allowed the onset of the competitive hydrolytic ring opening of HMF (yielding HHD) rather than its hydrogenation.

Calibration curves for the evaluation of conversion of HMF and products selectivity.

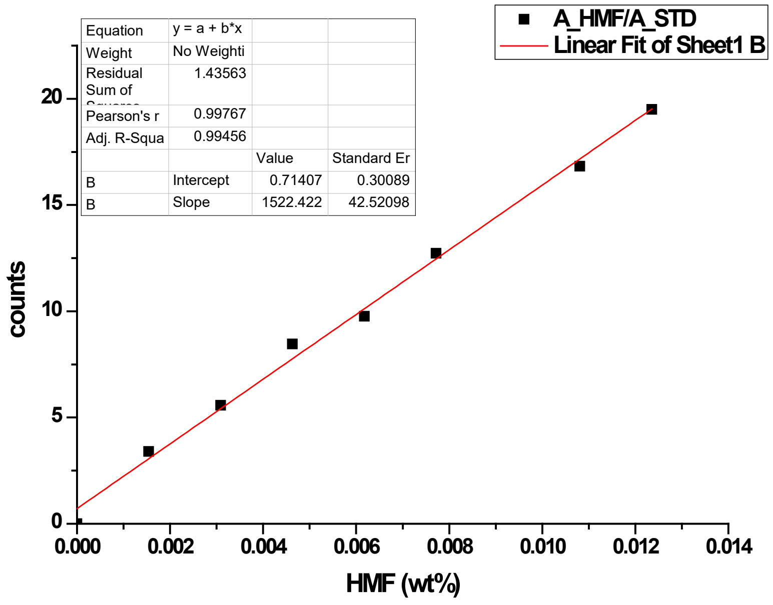

Figure S2. Calibration curve of HMF using diethyleneglycol dimethylether as a standard. [STD] = 0.01 M

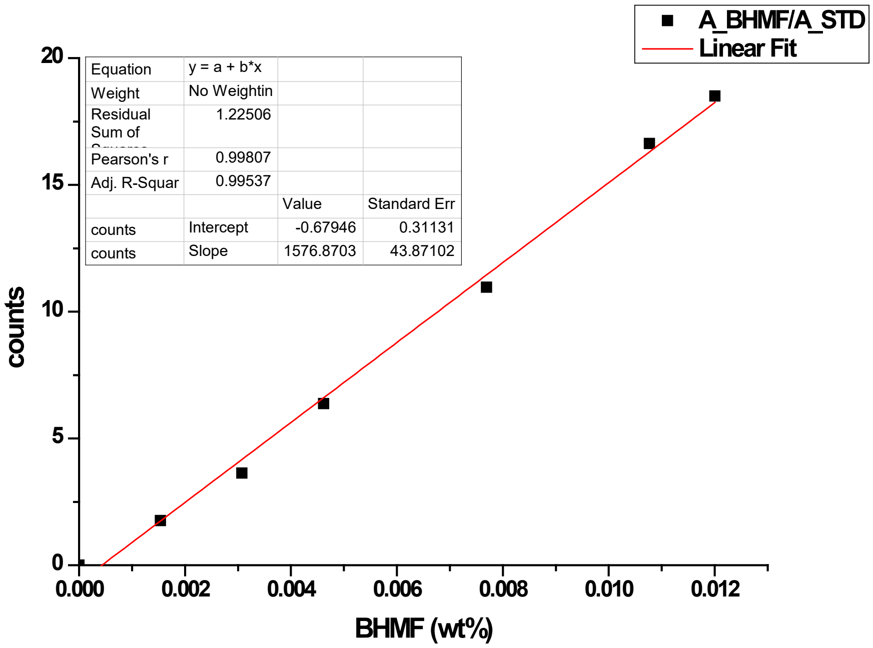

Figure S3. Calibration curve of BHMF using diethyleneglycol dimethylether as a standard. [STD] = 0.01 M

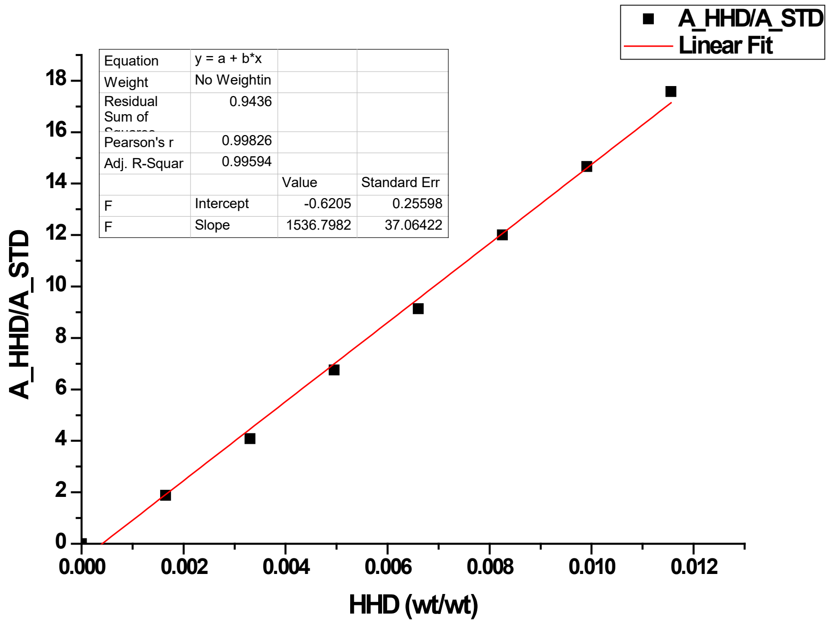

Figure S4. Calibration curve of HHD using diethyleneglycol dimethylether as a standard. [STD] = 0.01 M

The reaction of aqueous HMF at 60 °C.

Results are reported in Figure S5.

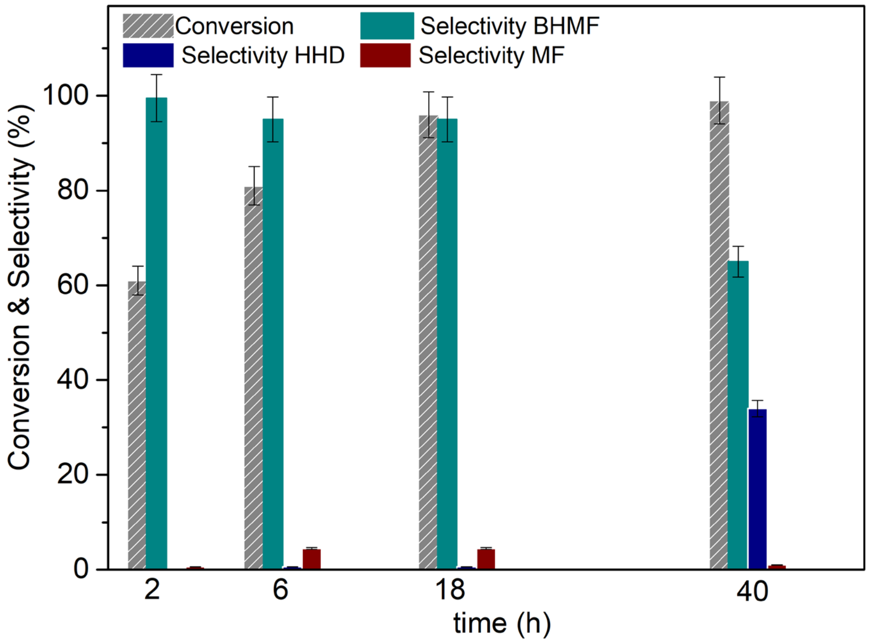

Figure S5. The effect of the reaction time on the batch reaction of HMF (autoclave). Conditions: 60 °C, $p(H_2)$ = 30 bar.

At 60 °C, the increase of the reaction time from 2 to 18 h increased the conversion as well from 62 to 96%, while the selectivity to BHMF was always steady to a very high value (≥95%). When the reaction was further prolonged up to 40 h, the process was still quantitative, but a significant presence of HHD (32%) was observed, with the consequence decrease of BHMF selectivity (64%). This indicated that the lowest investigated temperature not only led to the HMF ring opening, but it also corroborated the role of BHMF as an intermediate on the formation of HHD.

The multiphase reaction of HMF in a aqueous/isooctane biphase system

Results are summarized in Table S1.

Table S1. MP-reaction of HMF in a water-isooctane system.

Entry	T/p/t (ºC/bar/h)	Conversion (%)	Products (selectivity, %)		
			BMHF	MF	HHD
1	60/30/6	77	90	8	2
2	60/50/6	85	91	7	2
3	80/30/6	68	90	6	4
4	80/50/6	81	86	6	8

Conditions: p(H_2) = 30-50 bar, T= 60-80°C, t= 6 h, [HMF]= 0.2 M (10 mL) in deionized water, isooctane (5 mL), 5% Ru/C (50 mg).

Compared to the experiments performed using aq. HMF solutions, the addition of isooctane did not significantly alter the reaction outcome, except for a slight decrease of the conversion. This was consistent with the partitioning of the catalyst in the hydrocarbon phase, where HMF was not soluble. The segregation of Ru/C, however, was not observed plausibly because of a strong absorption (also via H-bonding interactions) of HMF and its products with the surface carboxylic and phenolic groups on the carbonaceous support of the catalyst, that partially retained Ru/C as a suspension in the aqueous phase. Attempts to induce the separation by acidifying the reaction mixture (to break down catalyst/reagent/products H-bonding) with extra aq. HCl until a pH=2 was reached, proved unsuccessful. The acidification helped the preferential partitioning of the catalyst in the hydrocarbon phase, but the overall separation was not yet satisfactory.

Synthesis of 2,5-bishydroxymethylfurane (BHMF)

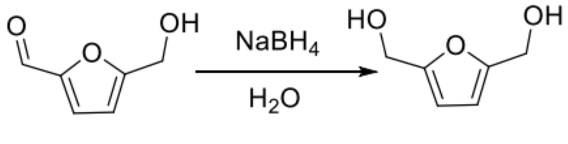

Scheme S1. The synthesis of BHMF by the stoichiometric reduction of HMF.

GC analyses confirmed that the conversion was complete, and the desired product was formed in a 97% amount. BHMF was extracted with ethyl acetate (4 x 15 mL). After solvent removal by rotary-evaporation, a white solid was obtained in a 97% purity (by GC) and 50% yield.

GC/MS (relative intensity, 70 eV) m/z: 128.0 (55), 111.0 (15), 109.0 (28), 97.0 (100), 69.1 (22), 55.0 (16), 53.1 (13), 44.0 (11), 41.0 (28). **^{1}H NMR in $CDCl_3$ (400 MHz) δ(ppm):** 9,57 (s, 1H, CHO), 7,22-7,21 (d, J (H,H) 3,7 Hz, 1H), 6,52- 6,50 (d, J (H,H) 3,6 Hz, 1H), 4,71 (s, 2H), 2,58 (s, 1H, OH). **^{13}C NMR** in $CDCl_3$ (100 MHz) δ(ppm): 154.2 (s, 2C), 108.72 (s, 2C), 57.68 (s, 2C).

Synthesis of Ionic Liquid Trioctylmetilphosphonium bis(trifluoro-methane)sulfonimide ($[P_{8881}][NTf_2]$)

$[P_{8,8,8,1}][NTf_2]$ was synthesized through a two-step procedure (A and B) reported by our group (Scheme S3a and S3b).[1] This is illustrated in the following two paragraphs.

Quaternarization of trioctyl phosphine with dimethylcarbonate to $[P_{8881}][MC]$

A mixture of trioctyl phosphine (20.8 g, 25 mL, 56 mmol), dimethylcarbonate (30 mL, 32.1 g, 356 mmol) methanol (30 mL) was placed in a 200 mL-jacketed sealed steel autoclave provided with a magnetic stirrer (Scheme 3A). The autoclave was closed and purged with three freeze pump-thaw cycles. This was very important to preserve trioctyl phosphine from oxidation. The mixture was then heated by oil circulation at 140 °C for 20 h.

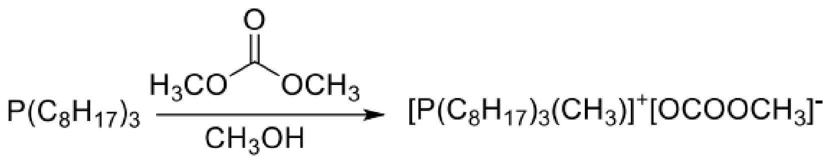

Scheme S2a. First step of the synthesis of $[P_{8,8,8,1}][NTf_2]$

After cooling to room temperature, residual DMC and methanol were removed by rotary evaporation and by high-vacuum pumping. Trioctylmetilphosphonium methylcarbonate ($[P_{8,8,8,1}][MeOCO_2]$) was obtained in a quantitative yield. The product was analysed by ^{1}H and ^{31}P NMR spectroscopy.

^{1}H NMR in DMSO-d6 (400 MHz) δ(ppm): 3,171 (s, 3H, CH3-OCOO), 2,152 (m, 6H), 1,799-1,764 (d, J (P,H)= 14,0 Hz, 3H, CH2-P), 1,466 (m, 6H), 1,373 (m, 6H), 1,274 (br m, 24H), 0,86 (t, 9H, CH3). **^{31}P NMR in DMSO-d6 (161 MHz) δ(ppm):** 32,30 (s, 1P).

Anion Exchange with $LiNTf_2$

In a 500-mL round-bottomed flask, commercial $LiNTf_2$ (16.75 g, 0,058 mol) was dissolved in milli-Q water (100 mL) and added to an aqueous solution of [$P_{8,8,8,1}$][$MeOCO_2$] (26.84 g, 0,058 mol in milli-Q water, 150 mL) (Scheme 13b).

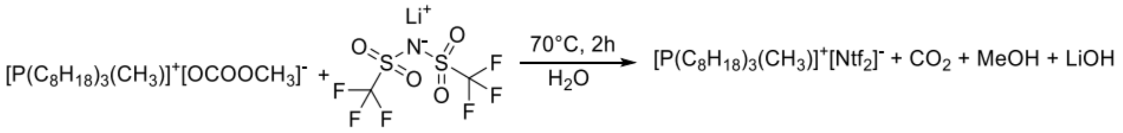

Scheme S2b. The second step of the synthesis of [$P_{8,8,8,1}$][NTf_2]

The reaction mixture was heated at 70 °C and kept under stirring for two hours. The homogeneous mixture rapidly turned to a white emulsion due to the formation of the desired product, [$P_{8,8,8,1}$][NTf_2], which was not soluble in water. After cooling to room temperature, extraction with CH_2Cl_2 (4x50 mL) was performed until the water phase became clear. The organic solvent was removed by rotary evaporation and high-vacuum pumping, yielding [P_{8881}][NTf_2] as a transparent viscous liquid in a quantitative yield. The product was analysed by 1H and 31P NMR spectroscopy.

^{1}H NMR in $CDCl_3$ (400 MHz) δ(ppm): 2,12 (m, 6H, CH2-P), 1,81-1,77 (d, J(P,H)= 13,2 Hz, 3H, CH3-P), 1,48 (m, 12H), 1,27 (br m, 24H), 0,88 (t, 9H, CH3). **^{31}P NMR in $CDCl_3$ (161 MHz) δ(ppm):** 31,39 (s, 1P).

General procedure for hydrogenation of Hydroxymethyl Furfural (HMF) in multiphasic batch system

Catalyst recycle procedure

At the end of a typical multiphase experiment, the aqueous phase was siphoned out of the glass reactor using a needle, under a moderate N_2 pressure. Fresh mill-Q water (10 mL) was added to wash the residual phases (Ru/C, isooctane and the IL). The system was stirred for 1 hour, after which water was replaced (by the siphoning method) with a fresh aq. HMF solution (10 mL). The multiphase mixture was then set to react according to the above-described procedure.

Leaching tests

Measures were performed using an Agilent 4210 MP-AES Microwave Plasma Atomic Emission Spectroscopy. The primary ionization excitation wavelength signal centred at λ= 372.8 nm was selected to detect Ru. A standard solution of $RuCl_3$ (1000 mg/L, HCl 10%) was diluted to prepare six aqueous solutions containing 0.05, 0.1, 0.25, 0.5, 1, 2.5 ppm of Ru; each one of these analysed by repeating the measure five times.
Thereafter, the aqueous sample A collected after recycling tests (Figure 5) from water/IL/iso-octane multiphase experiments, was analyzed. The detected amount of Ru was 0.01 ppm.
An aq. solution of HMF (0.05 M) was also analyzed as a blank.

Characterization of HHD

HHD was isolated after the reaction of HMF carried out at 100 °C, 50 bar, [HMF]= 0.05 M, 5% Ru/C (50 mg), 40 h (conditions of entry 3, Table 2). The isolation procedure was a modification/simplification of the method described by De Vries et al. [2] and was detailed in the body of the paper. The structure of the product was confirmed by GC/MS and NMR spectroscopy. **GC/MS (relative intensity, 70 eV) m/z:** 99,1 (100,0), 71,0 (16,0), 43,1 (98,0). **^{1}H NMR in $CDCl_3$ (400 MHz) δ(ppm):** 4,33 (s, 2H), 2,84 (t, 2H), 2,63 (t, 2H), 2,19 (s, 3H). **^{13}C NMR in $CDCl_3$ (100 MHz) δ(ppm):** 208,64 (s, 1C, C=O), 206,55 (s, 1C, C=O), 68,35 (s, 1C), 37,02 (s, 1C), 31,87 (s, 1C), 29,83 (s, 1C).

Hydrogenation of HMF in continuous flow mode

H-Cube® Mini Plus reactor used in this work, was comprised of:
- A hydrogen generator
- An HPLC pump that delivered either the solvents or the reaction mixtures inside the reactor, where they were mixed with hydrogen
- A computer to check the reaction temperature, the flow rate and the hydrogen pressure
- A packed column (CatCart®) containing the solid catalyst, where the reaction took place.
- A vial to collect the reaction mixture at the reactor outlet.

NMR characterization

Ionic liquid: Trioctylmetilphosphonium bis(trifluoro-methane)sulfonimide ([P_{8881}][NTf_2])

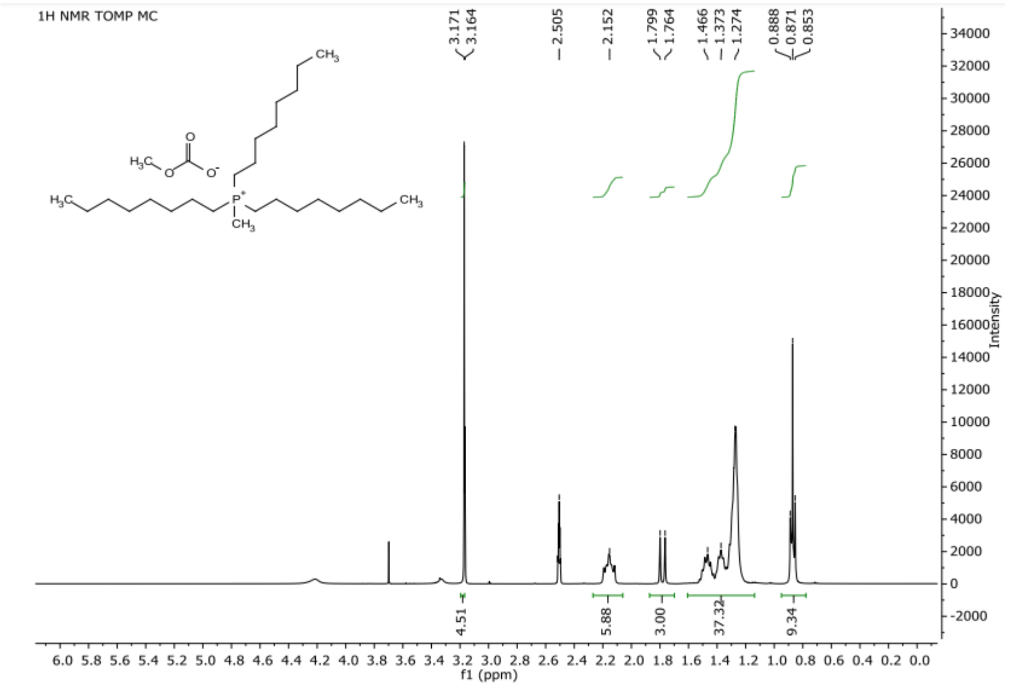

Figure S6. 1H NMR of [P_{8881}][MC] in DMSO-d6 (400 MHz) δ(ppm): 3.171 (s, 3H, CH3-OCOO), 2.152 (m, 6H), 1.799-1.764 (d, J (P,H)= 14.0 Hz, 3H, CH2-P), 1.466 (m, 6H), 1.373 (m, 6H), 1.274 (br m, 24H), 0.86 (t, 9H, CH3).

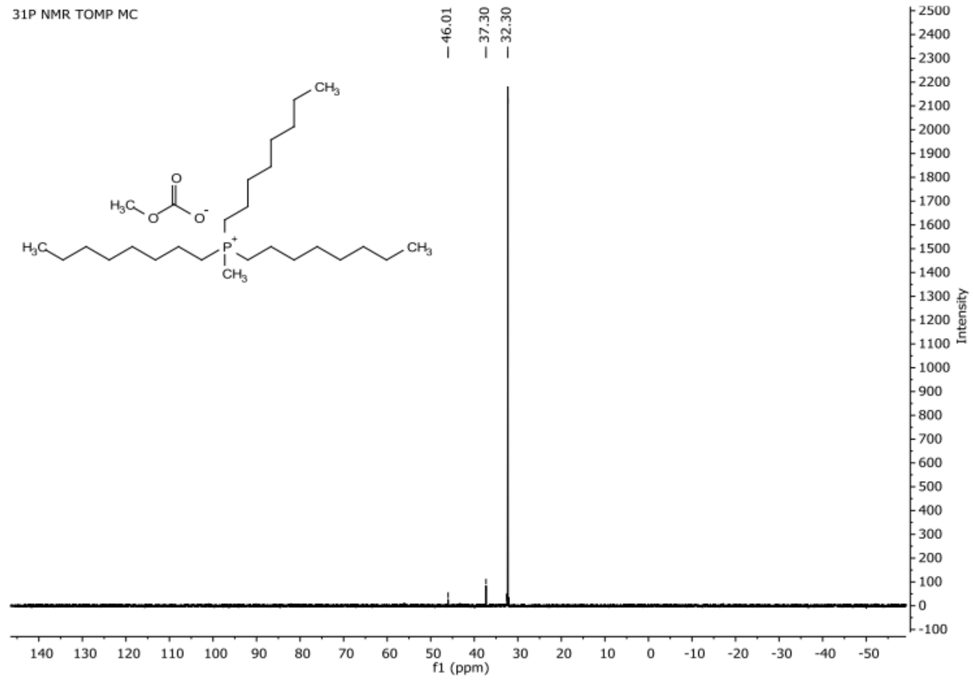

Figure S7. 31P NMR of [P_{8881}][MC] in DMSO-d6 (161 MHz) δ(ppm): 32.30 (s, 1P).

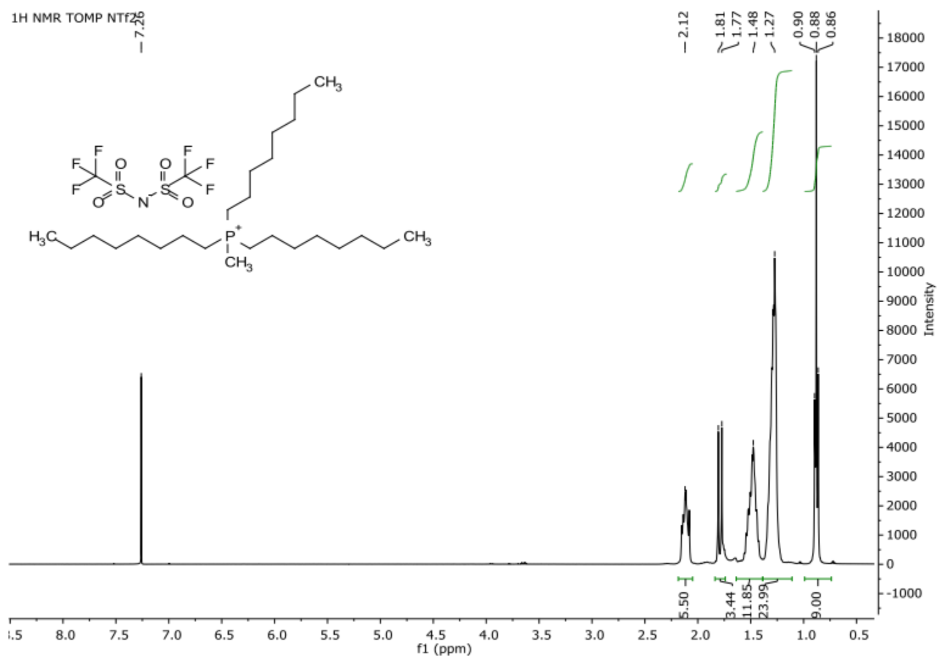

Figure S8. 1H NMR of [P8881][NTf2] in CDCl3 (400 MHz) δ(ppm): 2.12 (m, 6H, CH2-P), 1.81-1.77 (d, J(P,H)= 13.2 Hz, 3H, CH3-P), 1.48 (m, 12H), 1.27 (br m, 24H), 0.88 (t, 9H, CH3).

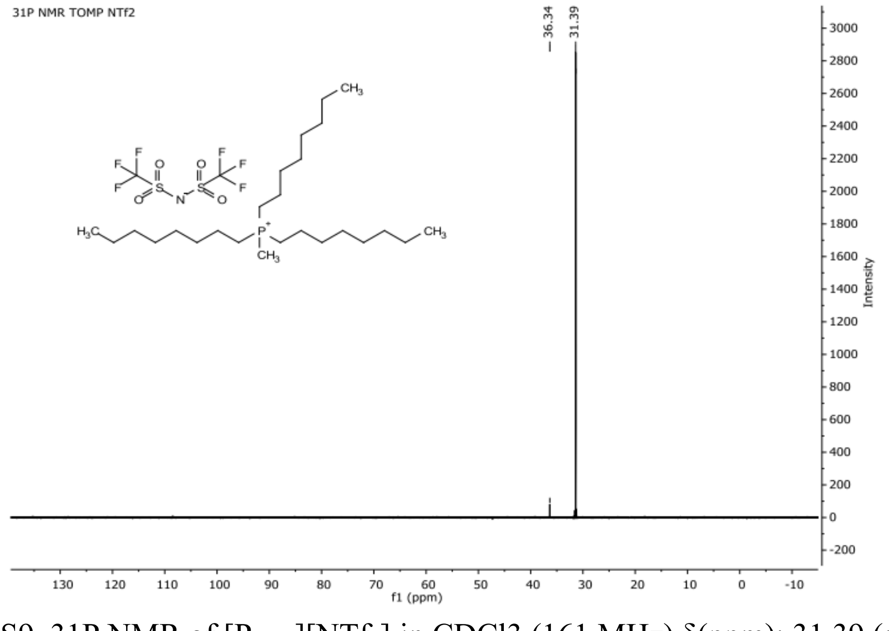

Figure S9. 31P NMR of [P8881][NTf2] in CDCl3 (161 MHz) δ(ppm): 31.39 (s, 1P).

BHMF (2,5-bis(hydroxymethyl)furan)

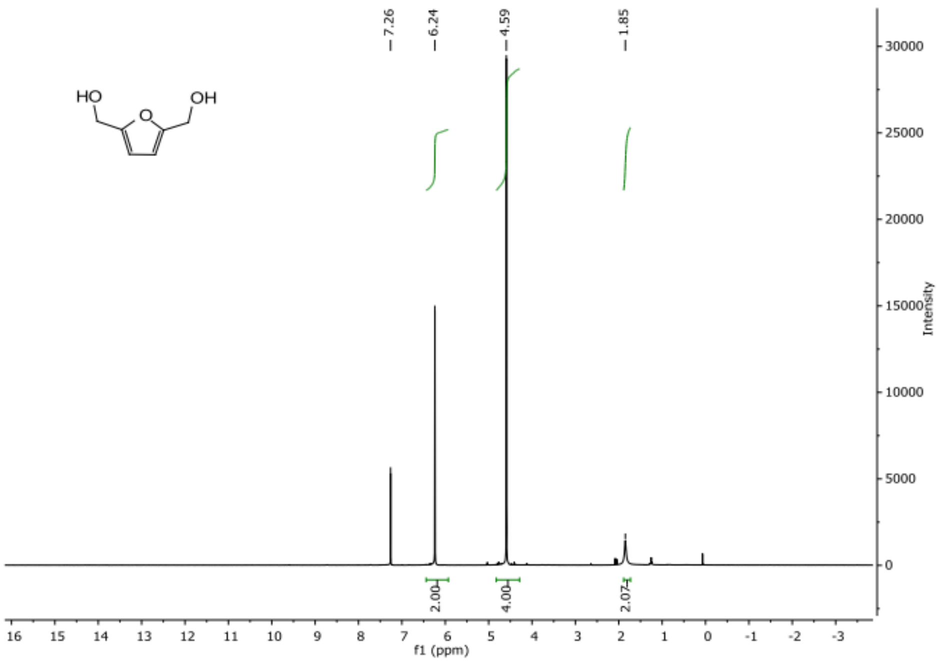

Figure S10. 1H NMR of BHMF in CDCl3 (400 MHz) δ(ppm): 6.24 (s, 2H), 4.59 (s, 4H), 2.07 (s, br, 2H).

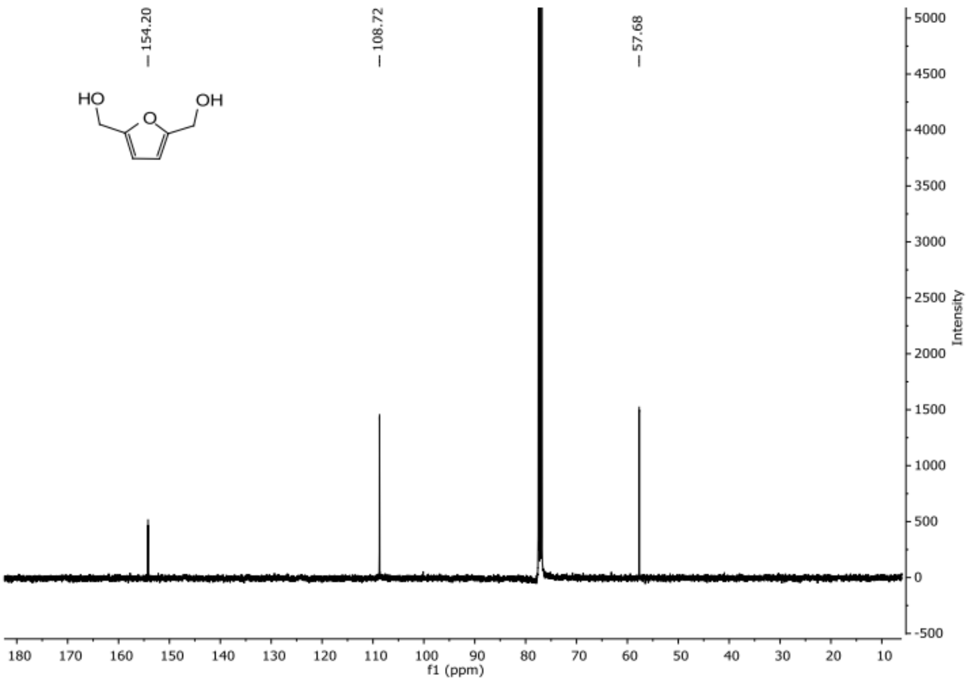

Figure S11. 13C NMR of BHMF in CDCl3 (100 MHz) δ(ppm): 154.2 (s, 2C), 108.72 (s, 2C), 57.68 (s, 2C).

DFF (2,5-diformylfurfural)

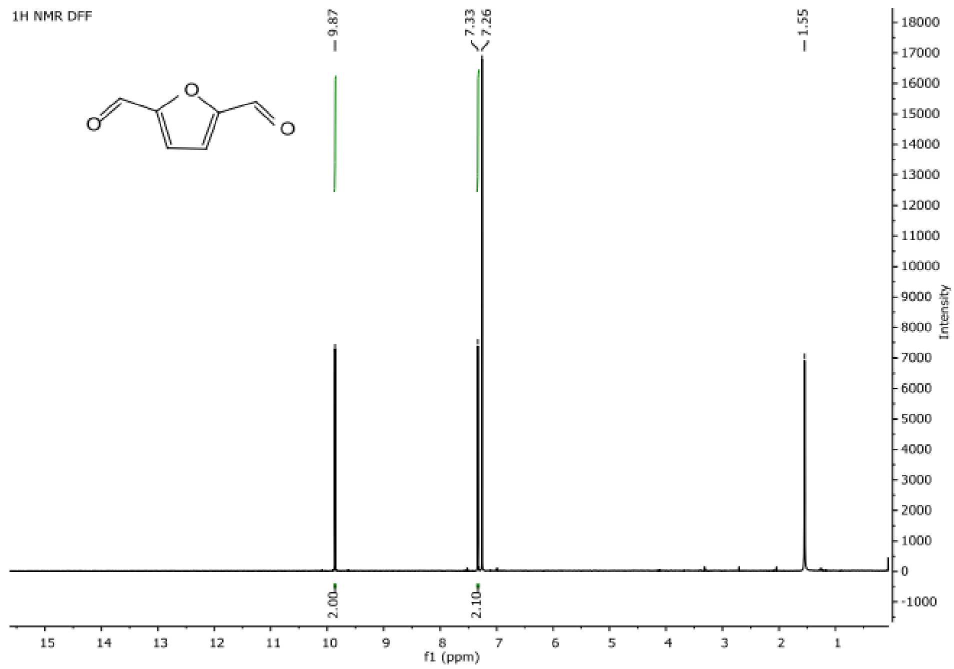

Figure S12. 1H NMR of DFF in CDCl3 (400 MHz)δ(ppm): 9.87 (s, 2H, CHO), 7.33 (s, 2H).

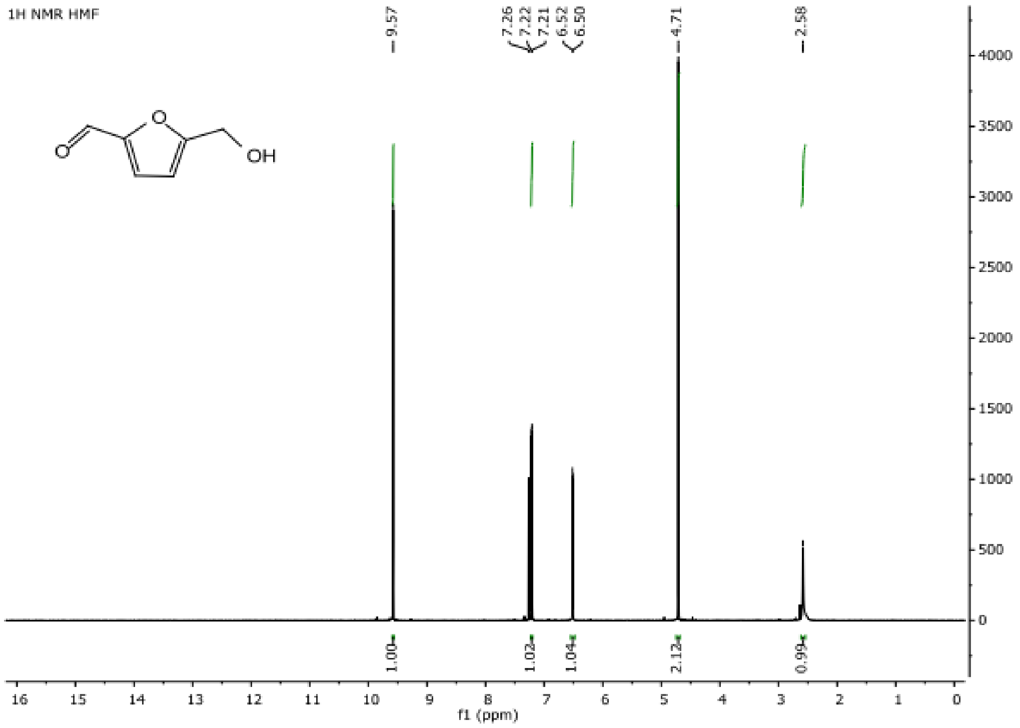

Figure S13. 1H NMR of HMF in CDCl3 (400 MHz)δ(ppm): 9.57 (s, 1H, CHO), 7.22 (d, JHH= 3.6Hz 1H), 6.52 (d, , JHH= 3.6Hz 1H), 4.71 (s, 2H), 2.58 (s, 1H, OH).

HHD (1-hydroxyhexane-2,5-dione)

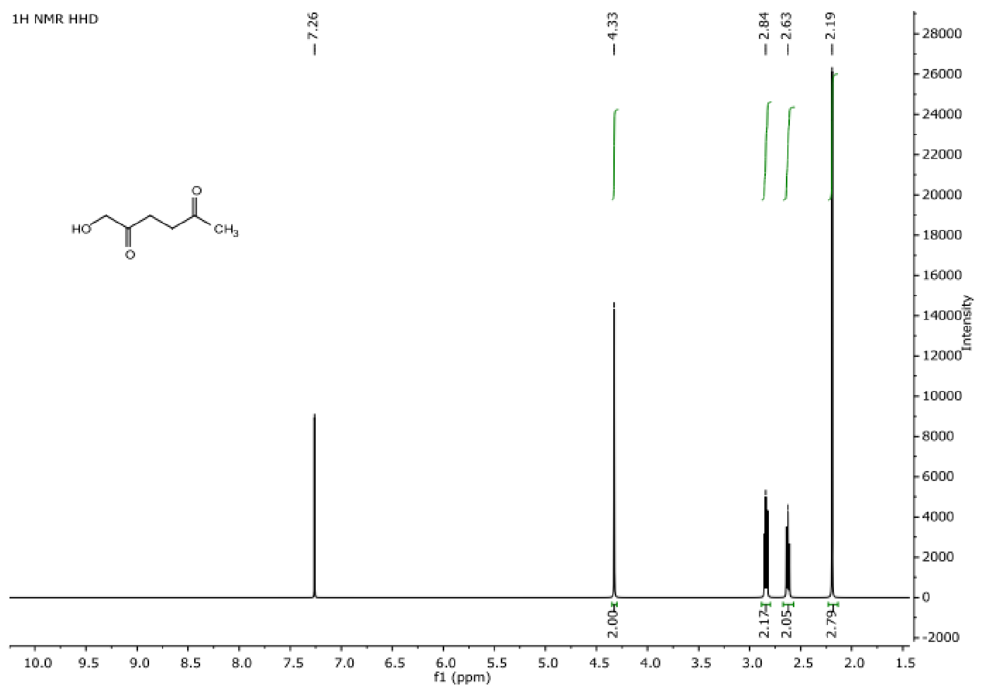

Figure S14. 1H NMR of HHD in CDCl3 (400 MHz) δ(ppm): 4.33 (s, 2H), 2.84 (t, 2H), 2.63 (t, 2H), 2.19 (s, 3H).

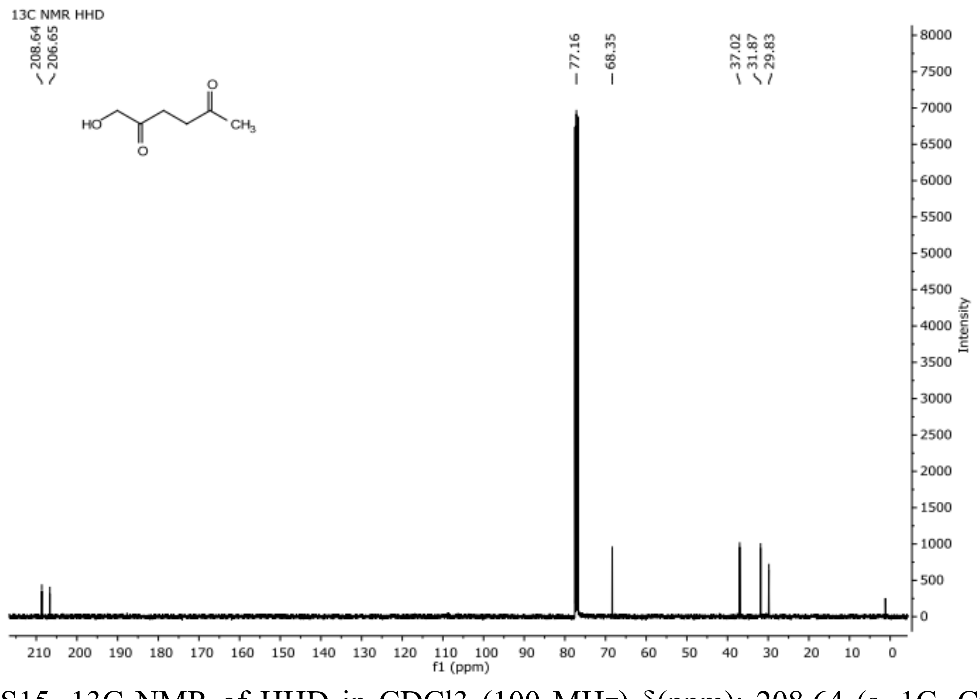

Figure S15. 13C NMR of HHD in CDCl3 (100 MHz) δ(ppm): 208,64 (s, 1C, C=O), 206.55 (s, 1C, C=O), 68.35 (s, 1C), 37.02 (s, 1C), 31.87 (s, 1C), 29.83 (s, 1C).

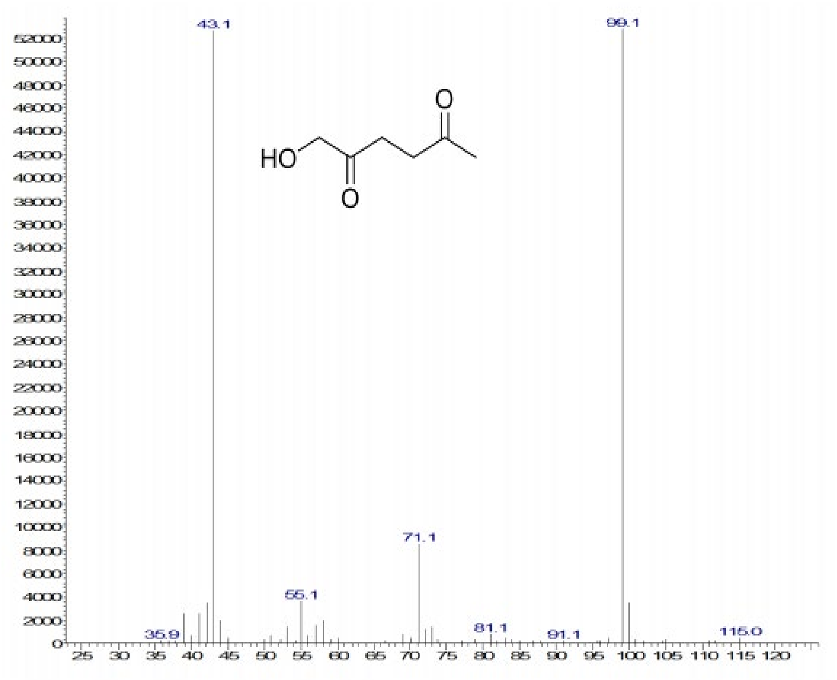

Figure S16. GC/MS of HHD (relative intensity, 70 eV) m/z: 99.1 (100.0), 71.0 (16.0), 43.1 (98.0).

Other Products

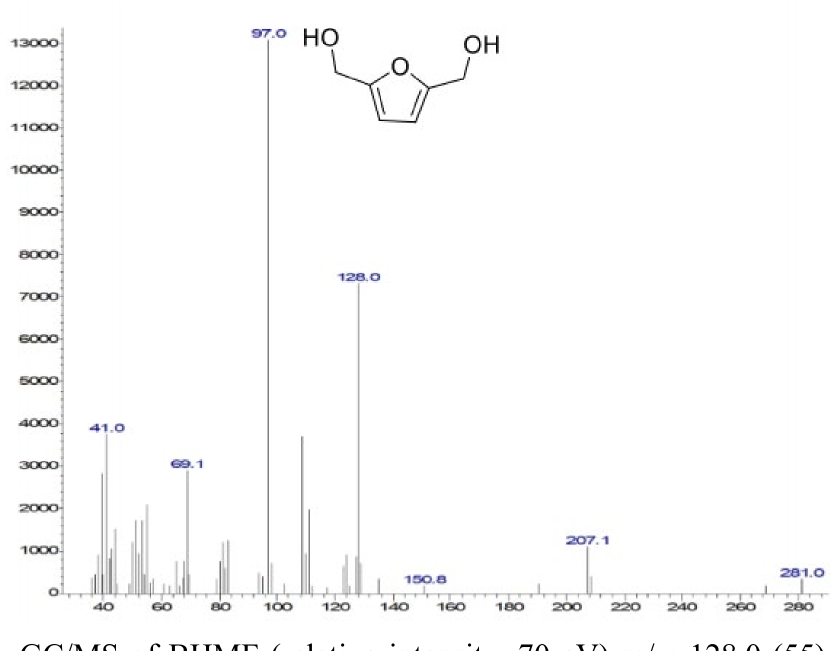

Figure S17. GC/MS of BHMF (relative intensity, 70 eV) m/z: 128.0 (55), 111.0 (15), 109.0 (28), 97.0 (100), 69.1 (22), 55.0 (16), 53.1 (13), 44.0 (11), 41.0 (28).

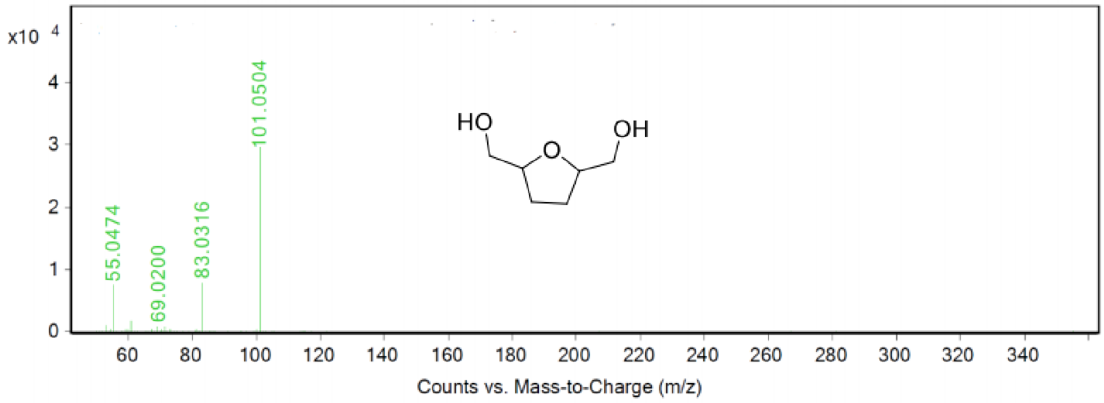

Figure 18. GC/MS of BHMTHF (relative intensity, 70 eV) m/z: 101 (100), 83 (26), 55 (25).

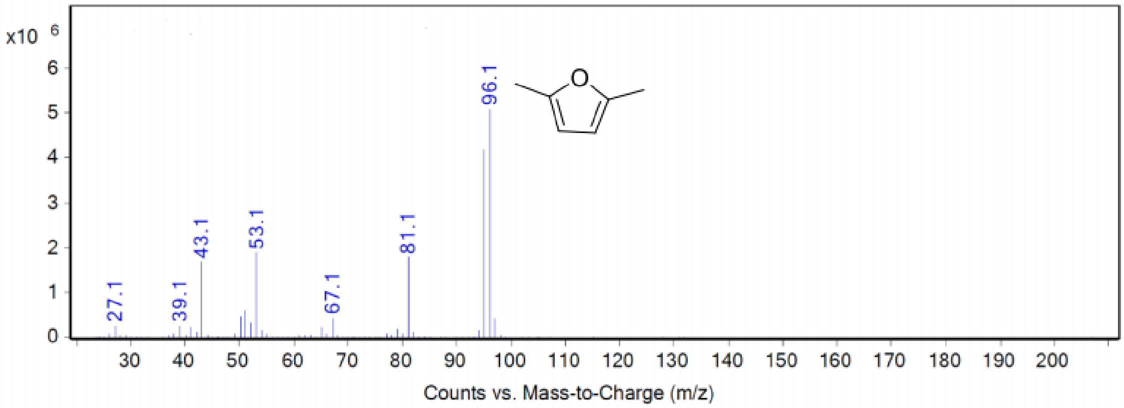

Figure 19. GC/MS of DMF (relative intensity, 70 eV) m/z: 96.1 (100), 95.1 (82), 81.1 (35), 53.1 (37), 43.1 (32).

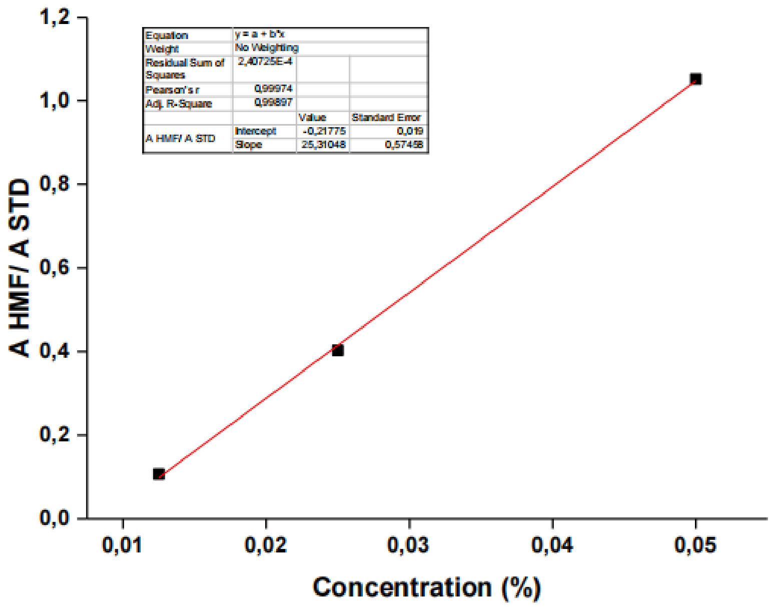

Figure S20. Calibration curve for the conversion of HMF with HP-5 capillary column (L=30 m, ø= 0,32 mm, film= 0,25 µm) with the method: 105°C for 2 min., 20°C/min, 150°C for 3 min., 25°C/min, 210°C for 3 min. Standard used: cyclohexane.

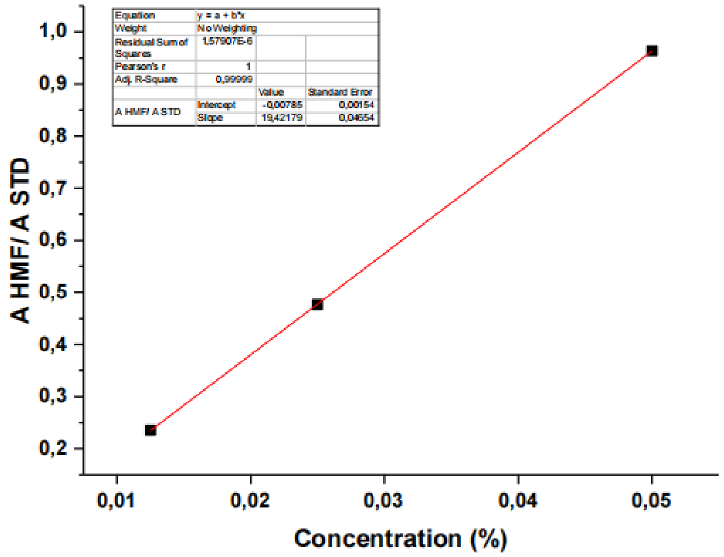

Figure S21. Calibration curve for the conversion of HMF with Restek Rt®-yDEXsa (L=30 m, ø= 0,25 mm, film= 0,25 μm) with the method: 60°C for 3 min., 20°C/min, 170°C for 10 min. Standard used: cyclohexane.

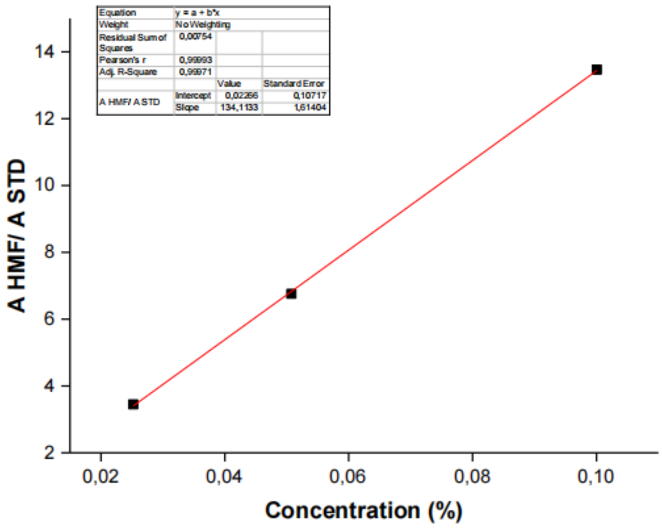

Figure S22. Calibration curve for the conversion of HMF with Perkin Elmer Elite 624 column, (L= 30 m, ø= 0,32 mm, film= 1,8 μm) with the method: 50 °C for 2 min., 25°C/min, 150°C for 15 min., 20°C/min, 240°C for 2 min. Standard used: diethyelene glycol dimethyl ether.

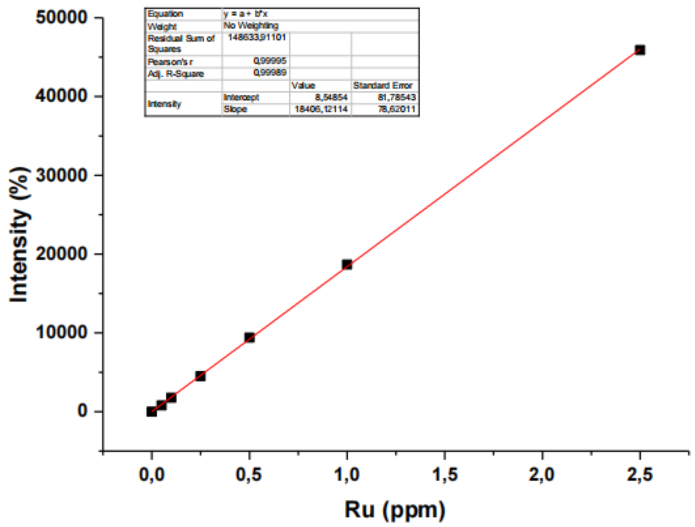

Figure S23. Calibration curve for Ru with MP-AES. Standard used: $RuCl_3$ in 10% HCl.

Continuous flow experiments

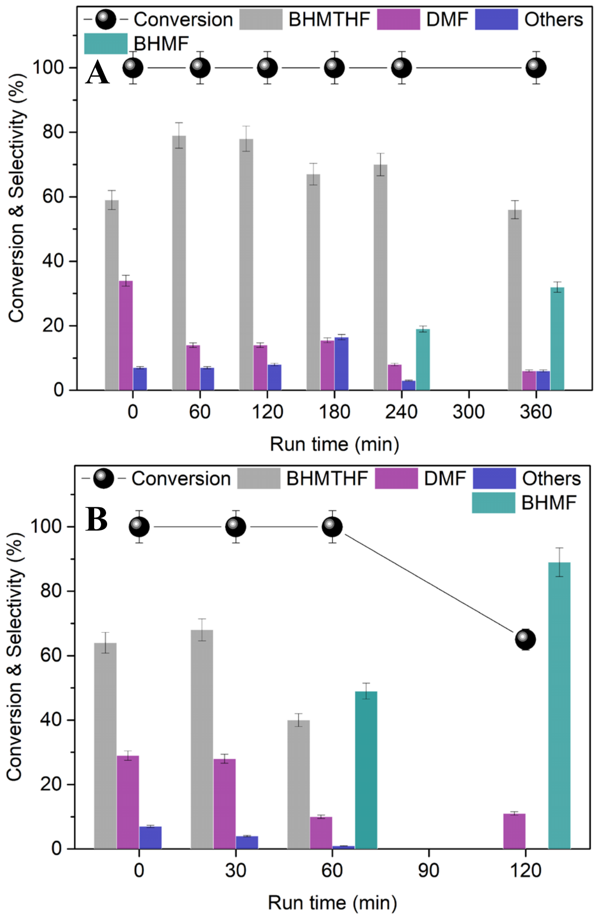

Figure S24. HMF hydrogenation in the continuous flow mode. Conditions: $p(H_2)$ = 50 bar, T= 100°C, HMF in EtOAc (0.025 M), Ru/C (0.3 g) in a CatCart® capsule, flow rate= 0.1 mL/min (A); flow rate= 0.3 mL/min (B). Conversion of HMF and products selectivity were obtained by GC-FID analysis.

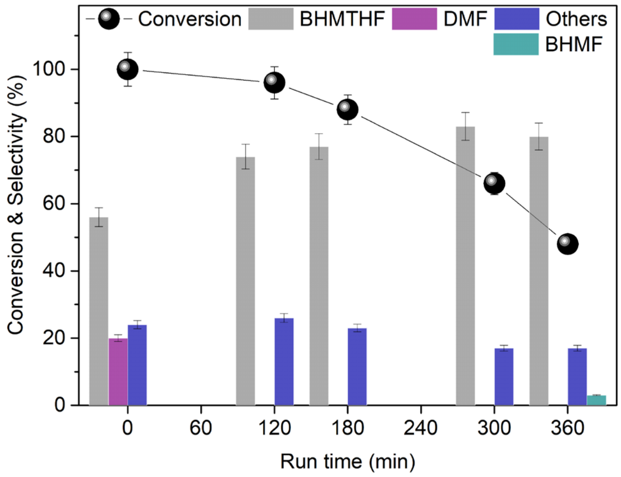

Figure S25. HMF hydrogenation in the continuous flow mode. Conditions: p(H_2) = 50 bar, T= 100°C, HMF in THF (0.05 M), Ru/C (0.3 g) in a CatCart® capsule, flow rate= 0.1 mL/min. Conversion of HMF and products selectivity were obtained by GC-FID analysis.

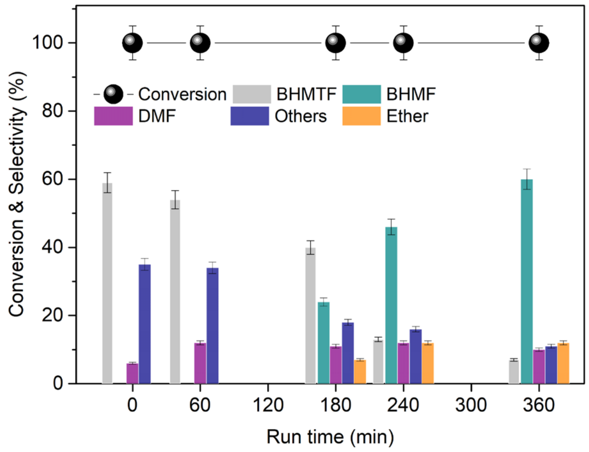

Figure S26. HMF hydrogenation in the continuous flow mode. Conditions: p(H_2)= 50 bar, T= 100°C, HMF in EtOH (0.05 M), Ru/C (0.3 g) in a CatCart® capsule, flow rate= 0.1 mL/min. Conversion of HMF and products selectivity were obtained by GC-FID analysis.

References

1. M. Fabris, V. Lucchini, M. Noè, A. Perosa, M. Selva, Ionic Liquids Made with Dimethyl Carbonate: Solvents as well as Boosted Basic Catalysts for the Michael Reaction, *Chem. Eur. J.*, **2009**, *15*, 12273–12282.
2. B. Wozniak, A. Spannenberg, Y. Li, S. Hinze, J.G. de Vries, *ChemSusChem*, **2018**, *11*, 356 – 359
